# Thermoelectric degrees of freedom determining thermoelectric efficiency

**DOI:** 10.1016/j.isci.2021.102934

**Published:** 2021-08-05

**Authors:** Byungki Ryu, Jaywan Chung, SuDong Park

**Affiliations:** 1 Energy Conversion Research Center, Electrical Materials Research Division, Korea Electrotechnology Research Institute (KERI), 12, Jeongiui-gil, Seongsan-gu, Changwon-si, Gyeongsangnam-do, 51543, Republic of Korea

**Keywords:** Thermal engineering, Electrical property, Thermal property, Electrical materials

## Abstract

For over half a century, the development of thermoelectric materials has based on the dimensionless figure of merit zT, assuming that the efficiency is mainly determined by this single parameter. Here, we show that the thermoelectric conversion efficiency is determined by *three* independent parameters, $Z_{\text{gen}}$, *τ*, and *β*, which we call the three thermoelectric degrees of freedom (DoFs). $Z_{\text{gen}}$ is the well-defined *mean* of the traditional zT under nonzero temperature differences. The two additional parameters *τ* and *β* are gradients of material properties and crucial to evaluating the heat current altered by nonzero Thomson heat and asymmetric Joule heat escape. Each parameter is a figure of merit. Therefore, increasing one of the three DoFs leads to higher efficiency. Our finding explains why the single-parameter theory is inaccurate. Further, it suggests an alternative direction in material discovery and device design in thermoelectrics, such as high *τ* and *β*, beyond zT.

## Highlights

• General efficiency theory of thermoelectric conversion is derived for *T*-dependent properties
• Three degrees of freedom Z_gen_, τ, and β determine thermoelectric efficiency
• Efficiency can vary up to approximately 40% with τ and β
• Each degree of freedom is a figure of merit

## Graphical abstract

**Figure:**
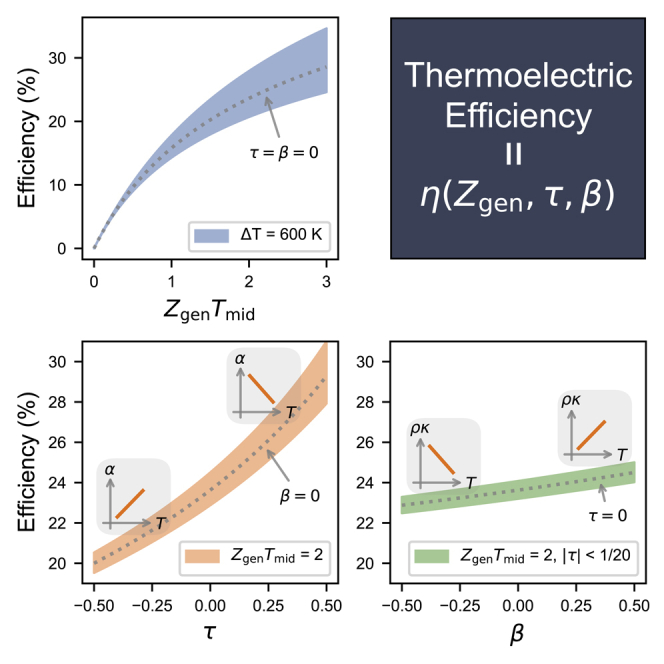

## Introduction

A thermoelectric device converts heat into electricity by generating a voltage in the thermoelectric leg placed between high-temperature $T_{h}$ and low-temperature $T_{c}$ regions through the Seebeck effect (Rowe (2005); Goupil (2015); Snyder and Toberer (2008)). A thermoelectric device, as a heat engine, has efficiency *η*, which is defined by the power *P* delivered to an external load divided by the amount of input heat current $Q_{h}$ flowing via heat diffusion and Peltier heat. When the thermoelectric properties are *constant*, the maximum thermoelectric efficiency $\eta_{\text{max}}$ of a thermoelectric material leg can be determined by the *single* parameter zT (Ioffe (1957)) defined as

$$zT:=\frac{\alpha^{2}}{\rho \kappa }T,$$

where the Seebeck coefficient *α*, electrical resistivity *ρ*, and thermal conductivity *κ* are three thermoelectric properties and *T* is the absolute temperature. In this *constant property model* (CPM), $\eta_{\text{max}}$ is expressed by the traditional efficiency equation, written as

(Equation 1) $$\eta_{\text{max}}^{\text{cpm}}=\frac{\Delta T}{T_{h}}\frac{\sqrt{1+zT_{\text{mid}}}-1}{\sqrt{1+zT_{\text{mid}}}+\frac{T_{c}}{T_{h}}},$$

where $\text{Δ}T:=T_{h}-T_{c}$ and $T_{\text{mid}}:=\left(T_{h}+T_{c}\right)/2$. With the observation that a high value of the dimensionless thermoelectric figure of merit $zT_{\text{mid}}$ gives a high $\eta_{\text{max}}$, many materials with low thermal conductivity and a high power factor ($\alpha^{2}$/*ρ*) have been successfully developed. As a result, the maximum value of the reported peak zT, which was less than 1 until 2000 (Heremans et al. (2013); Sales et al. (1996)), recently increased to approximately 3 (Zhao et al. (2014); Chang et al. (2018)).

However, the zT inaccurately predicts the efficiency because the thermoelectric properties greatly vary with *T* (Goupil (2015); Borrego Larralde (1961); Borrego (1964); Sherman et al. (1960); Sunderland and Burak (1964); Kim et al. (2015a, 2015b); Wee (2011); Ponnusamy et al. (2020a)). To improve the accuracy, various single parameters *averaging* the zT have been developed: the effective figure of merit $z_{\text{eff}}:=\frac{\langle \alpha \rangle^{2}}{\left\langle \rho \kappa \right\rangle }$ by Ioffe (Ioffe (1957)) and Borrego (Borrego Larralde (1961)), the engineering ZT (${\left[ZT\right]}_{\text{eng}}:=\frac{\langle \alpha \rangle^{2}}{\left\langle \rho \rangle \langle \kappa \right\rangle }\text{Δ}T$) by Kim et al. (Kim et al. (2015a)), and the modified figure of merit (${\left[ZT\right]}_{\text{mod}}$) by Min et al. (Min et al. (2004)), where the bracket ⟨⋅⟩ indicates averaging over *T*. But they are still inaccurate owing to a poor approximation of temperature gradient and/or an overestimation of Thomson heat (Ponnusamy et al. (2020b, 2020a)). While integrating zT or *z* over *T* is one of the most widely used averaging schemes, it severely overestimates the $\eta_{\text{max}}$ (Kim et al. (2015a)). Considering the entire zT curve may also not help. An example in the study by Ryu et al. (2020) provides two virtual materials in which one has a higher zT curve for the entire working temperature range but a lower efficiency than the other material. This thermoelectric paradox implies that *no* single-parameter scheme of averaging the zT curve can correct this reversed relation between the efficiency and zT.

To overcome the limitations of single-parameter theories, a few studies have been conducted by approximating the input and output heat currents at the leg thermal boundaries, $Q_{h}$ and $Q_{c}$. Min et al. (Min et al. (2004)) corrected the hot-side heat input of the CPM by adding half of the Thomson heat. More elaborate formalisms called *cumulative temperature-dependent* (CTD) property models were also suggested (Borrego (1964); Efremov and Pushkarsky (1971); Kim et al. (2015a)). However, the correction terms ruin the simplicity of the CPM and do not provide a figure of merit that is proportional to the efficiency. Furthermore, the reported CTD models do not improve the efficiency prediction accuracy, compared to the reported CPM theories. All the previous CPM and CTD theories have difficulty in predicting the performance of *advanced devices*, such as inhomogeneous materials and segmented/gradient legs, hindering the wide use of the efficiency prediction models in the electrical and thermal engineering fields.

## Results

**Figure 1:**
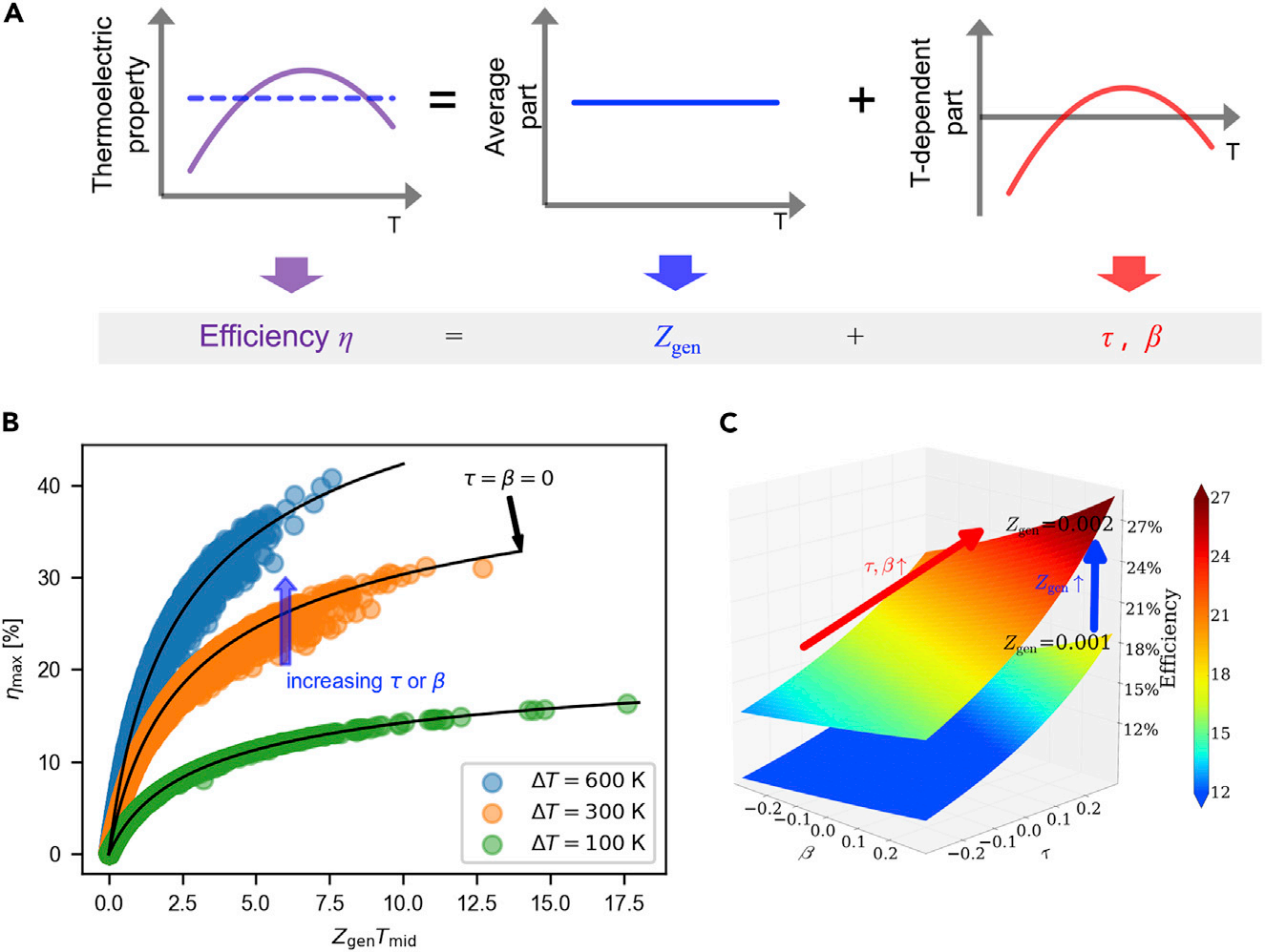
Efficiency and three thermoelectric DoFs (A) Schematic diagram explaining the relationship between temperature-dependent material properties and thermoelectric DoFs $Z_{\text{gen}}$, *τ*, and *β*. $Z_{\text{gen}}$ represents an average of material properties, while *τ* and *β* represent gradients of them. (B) Maximum efficiencies calculated for arbitrary quadratic thermoelectric property curves (see STAR Methods (efficiency computation, randomly generated thermoelectric properties) for the computational method and the arbitrary property generation method) having nonzero *τ* and *β*, with working temperature differences of ΔT=600K, 300K, and 100K and $T_{c}=300\;\text{K}$. Black solid lines correspond to the zero *τ* and *β* cases. (C) Efficiency surface $\eta_{\text{max}}^{\text{gen}}\left(Z_{\text{gen}},\beta ,\tau \right)$ in Equation 2 drawn for $Z_{\text{gen}}=0.002\;{\text{K}}^{-1}$ and $0.001\;{\text{K}}^{-1}$ for fixed $T_{h}=900\;\text{K}$ and $T_{c}=300\;\text{K}$. Improving one of the three thermoelectric DoFs $Z_{\text{gen}}$,*τ*, or *β* increases the efficiency.

In this paper, we show that the thermoelectric conversion efficiency is *completely determined* by *three* independent parameters $Z_{\text{gen}}$, *τ*, and *β*. Because they determine the performance of thermoelectric devices, we call them the *thermoelectric degrees of freedom* (DoFs). The schematic diagram in Figure 1A depicts the relationship between the material properties and the DoFs, where the temperature-dependent material properties are decomposed into average and gradient parts, and they are represented by the DoFs. $Z_{\text{gen}}$ is an average of material properties, and it generalizes the traditional figure of merit zT. The two additional DoFs *τ* and *β* are proportional to the escaped heat caused by the Thomson effect and the asymmetric Joule heat, respectively. The definitions of the DoFs will be given in the following section; see Equations 9, 10, and 14. The efficiency is a function of the three DoFs when the electric current *I* is given: $\eta =\eta \left(Z_{\text{gen}},\tau ,\beta |I\right)$. Furthermore, each DoF is a figure of merit because $\eta \left(Z_{\text{gen}},\tau ,\beta \right)$ is *monotonically* increasing in each variable. For a given $Z_{\text{gen}}$, the efficiency can vary by up to approximately 40% by virtue of the new figures of merit *τ* and *β* originating from the *T*-dependent properties; see Figure 1B.

The maximum efficiency can be simply approximated by the three DoFs:

(Equation 2) $$\eta_{\text{max}}=\eta_{\text{max}}\left(Z_{\text{gen}},\tau ,\beta |I=I_{\text{opt}}\right)\approx \eta_{\text{max}}^{\text{gen}}\left(Z_{\text{gen}},\tau ,\beta \right):=\frac{\Delta T}{T_{h}^{\text{′}}}\frac{\sqrt{1+Z_{\text{gen}}T_{\text{mid}}^{\text{′}}}-1}{\sqrt{1+Z_{\text{gen}}T_{\text{mid}}^{\text{′}}}+\frac{T_{c}^{\text{′}}}{T_{h}^{\text{′}}}},$$

where $I_{\text{opt}}$ is the optimal current giving the maximum efficiency,

(Equation 3) $$T_{h}^{\text{′}}:=T_{h}-\tau \Delta T,\;T_{c}^{\text{′}}:=T_{c}-\left(\tau +\beta \right)\Delta T,\;\text{and}\;T_{\text{mid}}^{\text{′}}:=\left(T_{h}^{\text{′}}+T_{c}^{\text{′}}\right)/2.$$

The general formula $\eta_{\text{max}}^{\text{gen}}$ is identical to the classical formula $\eta_{\text{max}}^{\text{cpm}}$ in Equation 1 except for the modification of the temperature parameters. While an exact computation of $Z_{\text{gen}}$, *τ*, and *β* requires the temperature distribution inside the device, they can be easily estimated using *one-shot approximation* assuming constant heat current and linear thermoelectric properties. The one-shot approximation gives

(Equation 4) $$\begin{array}{l} Z_{\text{gen}}\approx Z_{\text{gen}}^{\left(0\right)}:=\frac{{\left({\bar{\alpha }}^{\left(0\right)}\right)}^{2}}{{\left(\bar{\rho }\bar{\kappa }\right)}^{\left(0\right)}}=\frac{{\left(\frac{1}{\text{Δ}T}\int_{T_{c}}^{T_{h}}\alpha (T)dT\right)}^{2}}{\frac{1}{\text{Δ}T}\int_{T_{c}}^{T_{h}}\rho \left(T\right)\kappa \left(T\right)dT},\\ \tau \approx \tau_{\text{lin}}^{\left(0\right)}:=-\frac{1}{6}\frac{\alpha_{h}-\alpha_{c}}{{\bar{\alpha }}^{\left(0\right)}}=-\frac{1}{6}\frac{\alpha_{h}-\alpha_{c}}{\frac{1}{\text{Δ}T}\int_{T_{c}}^{T_{h}}\alpha (T)dT},\\ \beta \approx \beta_{\text{lin}}^{\left(0\right)}:=\frac{1}{6}\frac{{\left(\rho \kappa \right)}_{h}-{\left(\rho \kappa \right)}_{c}}{{\left(\bar{\rho }\bar{\kappa }\right)}^{\left(0\right)}}=\frac{1}{6}\frac{\rho_{h}\kappa_{h}-\rho_{c}\kappa_{c}}{\frac{1}{\text{Δ}T}\int_{T_{c}}^{T_{h}}\rho \left(T\right)\kappa \left(T\right)dT}, \end{array}$$

where the subscripts *h* and *c* indicate the material properties evaluated at $T_{h}$ and $T_{c}$, respectively. When the material properties are independent of temperature, the above formulas reduce to the CPM case: $\eta_{\text{max}}^{\text{gen}}=\eta_{\text{max}}^{\text{cpm}}$, $Z_{\text{gen}}=Z_{\text{gen}}^{\left(0\right)}=z$, $\tau =\tau_{\text{lin}}^{\left(0\right)}=0$, and $\beta =\beta_{\text{lin}}^{\left(0\right)}=0$. The simple formula in Equation 2 with Equation 4 predicts the maximum efficiency with high accuracy. The DoFs suggest new directions for improving the efficiency. Since each of $Z_{\text{gen}}$, *τ*, and *β* is a figure of merit, we can improve the efficiency by increasing one of them; see Figure 1C. Increasing the Seebeck coefficient on the cold side is a material optimization strategy, as it may increase *τ*. Segmenting a leg with different materials and modulating the DoFs is a device optimization strategy, increasing not only $Z_{\text{gen}}$ but also *τ* and *β*.

In the following sections, we sketch a derivation of the DoFs and explain why single-parameter theories are inexact for certain cases. Then, we give practical applications of the DoFs for optimization of materials and advanced devices.

### Thermoelectric DoFs in energy conversion

#### Temperature distribution

The thermoelectric effect is expressed in terms of electric current density *J* and heat current density $J^{Q}$:

$$\begin{array}{l} J=\sigma \left(E-\alpha \nabla T\right)\\ J^{Q}=\alpha TJ-\kappa \nabla T, \end{array}$$

where *E* is the electric field. Applying the charge and energy conservation laws to *J* and $J^{Q}$, we can obtain the thermoelectric differential equation of temperature T=T(x) in a one-dimensional thermoelectric leg (see, e.g., Chung and Ryu (2014); Goupil (2015)):

(Equation 5) $$\frac{d}{dx}\left(\kappa \frac{dT}{dx}\right)-T\left(\frac{d\alpha }{dT}\right)\left(\frac{dT}{dx}\right)J+\rho J^{2}=0,$$

where *x* is the spatial coordinate inside the leg. The left-hand side of Equation 5 is composed of thermal diffusion, Thomson heat generation, and Joule heat generation. Here, we obtain an *integral equation* for T(x) by integrating Equation 5 twice. Let $f_{T}\left(x\right):=-T\frac{d\alpha }{dT}\frac{dT}{dx}J+\rho J^{2}$ be the heat source term of Equation 5. If we *assume* that κ(x) and $f_{T}\left(x\right)$ are known, the Equation 5 becomes linear. Then, the Equation 5 with Dirichlet boundary conditions

(Equation 6) $$T\left(0\right)=T_{h},\;T\left(L\right)=T_{c}$$

can be solved to find an integral equation for *T* and $\frac{dT}{dx}$:

(Equation 7) $$\begin{array}{l} T\left(x\right)=\left(T_{h}-\frac{K\text{Δ}T}{A}\int_{0}^{x}\frac{1}{\kappa \left(s\right)}\;ds\right)+\left(-\int_{0}^{x}\frac{F_{T}\left(s\right)}{\kappa \left(s\right)}\;ds+\frac{K\;\delta T}{A}\int_{0}^{x}\frac{1}{\kappa \left(s\right)}\;ds\right),\\ \frac{dT}{dx}\left(x\right)=\left(-\frac{K\text{Δ}T}{A}\frac{1}{\kappa \left(x\right)}\right)+\left(\frac{F_{T}\left(x\right)}{\kappa \left(x\right)}+\frac{K\;\delta T}{A}\frac{1}{\kappa \left(x\right)}\right), \end{array}$$

where $F_{T}\left(x\right):=\int_{0}^{x}\;f_{T}\left(s\right)\;ds$ and $\delta T:=\int_{0}^{L}\frac{F_{T}\left(x\right)}{\kappa \left(x\right)}\;dx$. For a detailed derivation, see STAR Methods (Integral equations of T(x) and dTdx(x)) and Figure S1. The integral equations are of the form $T=\varphi \left[T\right]$, where *φ* is an integral operator. With this relation, the exact *T* can be obtained via fixed-point iteration $T_{n+1}=\varphi \left[T_{n}\right]$ (see, e.g., (Burden and Faires, 2010, Section 2.2)). The detailed procedure to find T(x) can be found in STAR Methods (numerical method for finding temperature solution). An approximate *T* can be obtained from $T=\varphi \left[T^{\left(0\right)}\right]$, where $T^{\left(0\right)}$ is the solution of Equation 5 when J=0. Once the temperature distribution *T* is found, the thermoelectric performance and efficiency can be easily computed.

#### Electrical power and $Z_{\text{gen}}$

To compute the open-circuit voltage *V*, electrical resistance *R*, and thermal resistance 1/K, integrating the material properties over the spatial coordinate *x* inside the leg (not over *T*) is natural because the electric current and the heat current flow through the leg. Hence, we define the average parameters of the material properties as

(Equation 8) $$\begin{array}{l} \bar{\alpha }:=\frac{V}{\text{Δ}T}=\frac{1}{\text{Δ}T}\int \left(-\alpha \frac{dT}{dx}\right)\;dx,\\ \bar{\rho }:=\frac{A}{L}R\;=\frac{1}{L}\int \rho \;dx,\\ \frac{1}{\bar{\kappa }}:=\frac{A}{L}\frac{1}{K}=\frac{1}{L}\int \frac{1}{\kappa }\;dx, \end{array}$$

where *L* and *A* are the length and area of the leg. Here, $\bar{\alpha }$ is independent of T(x) if $T_{h}$ and $T_{c}$ are fixed and the material is homogeneous and isotropic. For an additional discussion on device parameters, see STAR Methods (device parameters) and Figure S2. The electric current is determined as $I=\frac{V}{R\left(1+\gamma \right)}$, where *γ* is the ratio of the load resistance $R_{\text{L}}$ outside the device to the resistance of the thermoelectric leg *R*; see STAR Methods (electric current equation). The power *P* delivered to the load is given as

$$P=I^{2}R_{\text{L}}=I\left(V-IR\right)=\frac{{\bar{\alpha }}^{2}}{\bar{\rho }}\frac{\text{Δ}T^{2}}{L/A}\frac{\gamma }{{\left(1+\gamma \right)}^{2}},$$

which is maximized near γ=1. Using the average properties, we define the *general device power factor* $PF_{\text{gen}}$ as

$$PF_{\text{gen}}:=\frac{{\bar{\alpha }}^{2}}{\bar{\rho }}.$$

From this, the *general device figure of merit* is defined as

(Equation 9) $$Z_{\text{gen}}:=\frac{{\left(V/\text{Δ}T\right)}^{2}}{RK}=\frac{{\bar{\alpha }}^{2}}{\bar{\rho }\;\bar{\kappa }}.$$

This generalizes the classical device figure of merit. If the material properties are temperature-independent, the $Z_{\text{gen}}$ is reduced to the conventional material parameter *z*.

#### Heat current, *τ* and *β*

If the material properties do not depend on *T*, then the heat currents on the hot and cold sides are determined by the average parameters:

$$\begin{array}{c} \bar{Q_{h}}=K\text{Δ}T+I\bar{\alpha }T_{h}-\frac{1}{2}I^{2}R,\\ \bar{Q_{c}}=K\text{Δ}T+I\bar{\alpha }T_{c}+\frac{1}{2}I^{2}R. \end{array}$$

The power delivered to the outside is $P=\bar{Q_{h}}-\bar{Q_{c}}=I\bar{\alpha }\text{Δ}T-I^{2}R=I\left(V-IR\right)$. In contrast, if the material properties *depend on T*, then the heat currents may change to other values $Q_{h}$ and $Q_{c}$. However, if the average parameters $\bar{\alpha }$ and $\bar{\rho }$ remain unchanged for each *I*, then the difference between the hot- and cold-side heat currents remains unchanged, indicating that the power remains the same: $P=Q_{h}-Q_{c}=\bar{Q_{h}}-\bar{Q_{c}}$. Thus, we have the relation $P=Q_{h}-Q_{c}=\left(\bar{Q_{h}}-B\right)-\left(\bar{Q_{c}}-B\right)$ for some *B*, implying that both of the heat currents are shifted by the same *backward heat current B*. We can show that *B* is determined by the newly introduced gradient parameters *τ* and *β* as $B=\left(I\bar{\alpha }\text{Δ}T\right)\tau +\left(\frac{1}{2}I^{2}R\right)\beta$; see STAR Methods (heat current and two additional DoFs τ and β). The definitions of *τ* and *β* will be given soon in Equation 14. From this relation between *B*, *τ,* and *β*, the heat currents on the hot and cold sides are determined as

(Equation 10) $$Q_{h}=K\text{Δ}T+I\bar{\alpha }\left(T_{h}-\tau \text{Δ}T\right)-\frac{1}{2}I^{2}R\left(1+\beta \right),\;Q_{c}=K\text{Δ}T+I\bar{\alpha }\left(T_{c}-\tau \text{Δ}T\right)+\frac{1}{2}I^{2}R\left(1-\beta \right).$$

Note that the heat currents are altered by the effective Thomson heat flow $\left(-I\bar{\alpha }\text{Δ}T\right)\tau$ and the asymmetric Joule heat escape $\left(-\frac{1}{2}I^{2}R\right)\beta$ due to the temperature dependence.

The exact formulations of *τ* and *β* can be obtained by using the second equation in Equation 7 for $\frac{dT}{dx}\left(x\right)$. First, we derive an integral equation of the heat currents in the form of

(Equation 11) $$\begin{array}{l} Q_{h}=I\alpha_{h}T_{h}-A\kappa_{h}{\left(\frac{dT}{dx}\right)}_{h}=I\alpha_{h}T_{h}+K\left(\Delta T-\delta T\right),\\ Q_{c}=I\alpha_{c}T_{c}-A\kappa_{c}{\left(\frac{dT}{dx}\right)}_{c}=Q_{h}-P. \end{array}$$

δT has two contribution terms of *I* and $I^{2}$ from the double integration of Thomson and Joule heat; since

(Equation 12) $$\begin{array}{c} F_{T}\left(x\right)=I^{2}\int_{0}^{x}\frac{1}{A^{2}}\;\rho \left(s\right)\;ds-I\int_{0}^{x}\frac{1}{A}T\left(s\right)\cdot \frac{d\alpha }{dT}\left(T\left(s\right)\right)\cdot \frac{dT}{dx}\left(s\right)\;ds\;\\ =:\;I^{2}F_{T}^{\left(2\right)}\left(x\right)-IF_{T}^{\left(1\right)}\left(x\right), \end{array}$$

we have

(Equation 13) $$\begin{array}{c} \delta T=I^{2}\int_{0}^{L}\frac{F_{T}^{\left(2\right)}\left(x\right)}{\kappa \left(x\right)}\;dx-I\int_{0}^{L}\frac{F_{T}^{\left(1\right)}\left(x\right)}{\kappa \left(x\right)}\;dx\\ =:I^{2}\delta T^{\left(2\right)}-I\delta T^{\left(1\right)}. \end{array}$$

Rewriting the $Q_{h}$ in Equation 11 into the form of Equation 10, we obtain

(Equation 14) $$\begin{array}{l} \tau :=\frac{1}{\bar{\alpha }\text{Δ}T}\left[\left(\bar{\alpha }-\alpha_{h}\right)T_{h}-K\;\delta T^{\left(1\right)}\right],\\ \beta :=\frac{2}{R}K\;\delta T^{\left(2\right)}-1. \end{array}$$

For *T*-independent material properties, $\delta T^{\left(2\right)}=\frac{1}{2}\frac{R}{K}$ and $\delta T^{\left(1\right)}\equiv 0$ so τ≡0 and β≡0, which implies $Q_{h}=\bar{Q_{h}}=K\text{Δ}T+I\bar{\alpha }T_{h}-\frac{1}{2}I^{2}R$, as expected.

**Figure 2:**
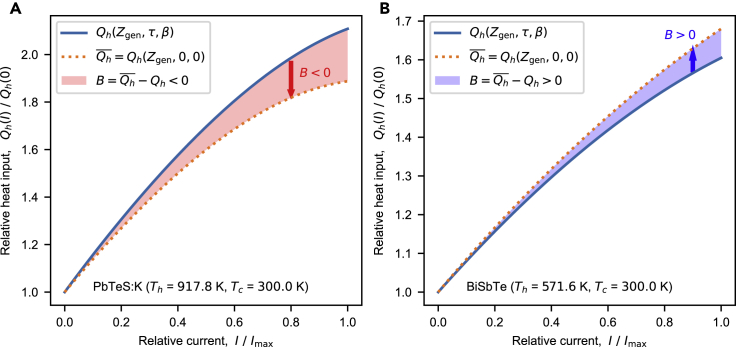
Heat and electric currents: $Q_{h}\left(I\right)$ vs. *I* (A) Relative hot-side heat currents for PbTeSe:K (Wu et al. (2014c)) with and without *τ* and *β* ($Q_{h}$ and $\bar{Q_{h}}$). (B) Relative hot-side heat currents for BiSbTe (Son et al. (2013)) with and without *τ* and *β* ($Q_{h}$ and $\bar{Q_{h}}$). The shaded regions represent the *backward heat*$B=\bar{Q_{h}}-Q_{h}=\bar{Q_{c}}-Q_{c}$: red color for negative values and blue color for positive values.

Figure 2 shows the heat and electric currents of one-dimensional single-leg thermoelectric generators using PbTeSe:K (Wu et al. (2014c)) and BiSbTe (Son et al. (2013)). With increasing *I* from 0 to the maximum current $I_{\text{max}}$ where V−IR=0, both $Q_{h}$ and $\bar{Q_{h}}$ have the same increasing tendency, mainly owing to the Peltier effect. However, their discrepancy $B=\bar{Q_{h}}-Q_{h}$ increases with *I*. In CPM-like models where τ=β=0, which corresponds to Goldsmid's device figure of merit (Goldsmid (1960)), the $\bar{Q_{h}}$ at $I_{\text{max}}$ is underestimated by 10% for PbTeSe:K and overestimated by 5% for BiSbTe. This indicates that a temperature dependency can significantly affect the thermoelectric performance, causing a large discrepancy in the thermoelectric performance between the exact model and CPM.

#### Thermoelectric efficiency and DoFs

The thermoelectric efficiency is defined as $\eta :=\frac{Q_{h}-Q_{c}}{Q_{h}}$. From Equation 10, we can verify that the dimensionless heat currents, $\frac{Q_{h}}{K\text{Δ}T}$ and $\frac{Q_{c}}{K\text{Δ}T}$, and the efficiency are determined by the five parameters $Z_{\text{gen}}$, *τ*, *β*, ΔT, and *γ* (or *I*). Therefore, given external thermal and electrical conditions, the efficiency is *exactly* determined by the three DoFs as

(Equation 15) $$\eta \left(Z_{\text{gen}},\tau ,\beta |T_{h},T_{c},\gamma \right)=\frac{\Delta T\frac{\gamma }{{\left(1+\gamma \right)}^{2}}}{\frac{1}{Z_{\text{gen}}}+\left(\frac{1}{1+\gamma }\right)\left(T_{h}-\tau \Delta T\right)-\frac{1}{2}\Delta T{\left(\frac{1}{1+\gamma }\right)}^{2}\left(1+\beta \right)}.$$

**Figure 3:**
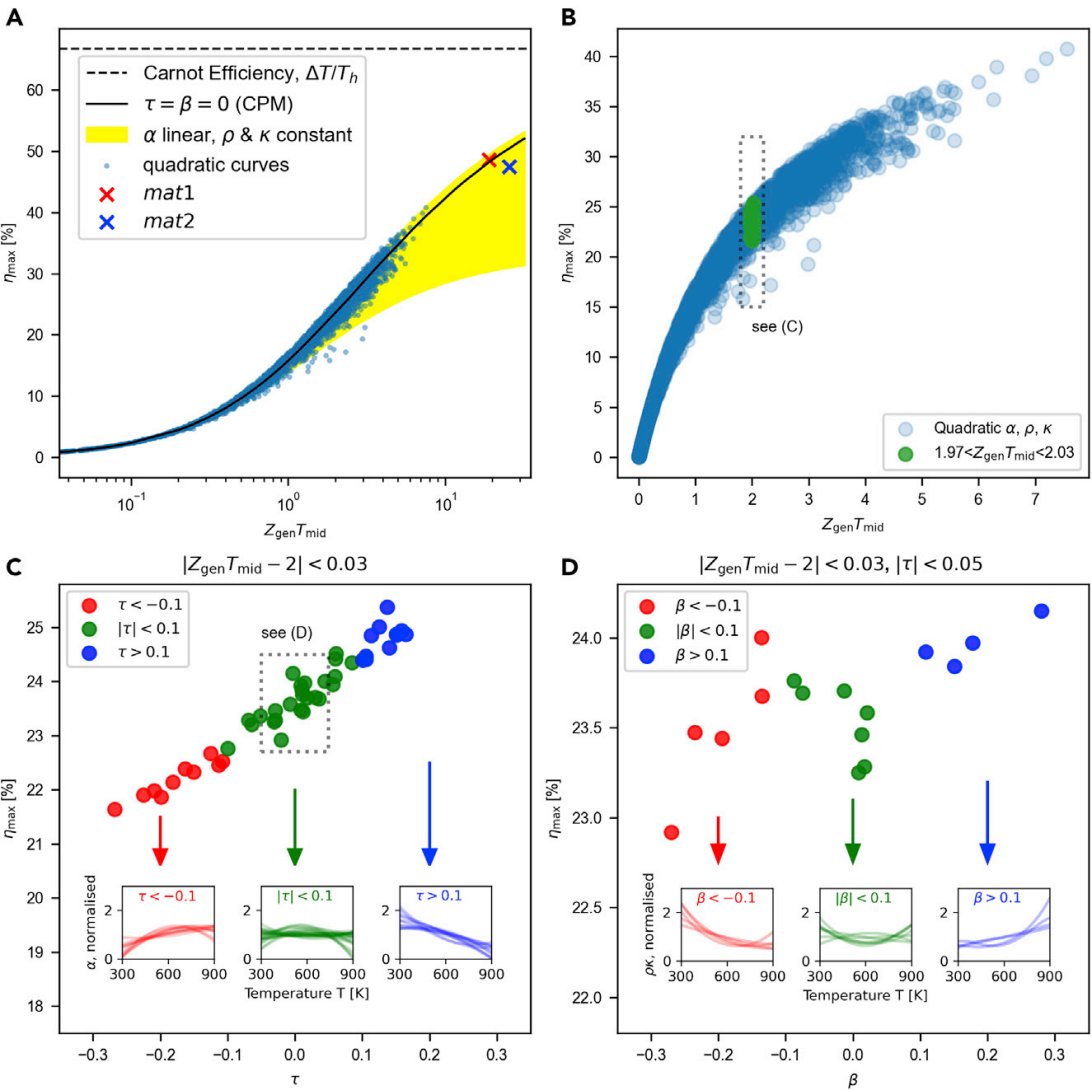
$\eta_{\text{max}}$ and three thermoelectric DoFs when $T_{c}=300\;\text{K}$ and $T_{h}=900\;\text{K}$ (A) $\eta_{\text{max}}$ vs. $Z_{\text{gen}}T_{\text{mid}}$ for various thermoelectric curves: CPM (black solid line), linear *α* and contant *ρ* and *κ* case (yellow shaded), and arbitrary quadratic thermoelectric property curves (blue dots; see STAR Methods (randomly generated thermoelectric properties) for the raw data). *mat1* (red cross) and *mat2* (blue cross) from Ryu et al. (2020). (B) $\eta_{\text{max}}$ and $Z_{\text{gen}}T_{\text{mid}}$ for the quadratic curves. (C) $\eta_{\text{max}}$ and *τ* when $\left|Z_{\text{gen}}T_{\text{mid}}-2\right|<0.03$. (D) $\eta_{\text{max}}$ and *β* when $\left|Z_{\text{gen}}T_{\text{mid}}-2\right|<0.03$ and |τ|<0.05.

For a detailed derivation, see STAR Methods (thermoelectric efficiency has three degrees of freedom). Similar to other heat engines, the efficiency of thermoelectric modules increases with respect to ΔT, as shown in Figure 1B. Furthermore, for fixed ΔT and *γ*, the efficiency monotonically increases with respect to $Z_{\text{gen}}$, *τ*, and *β*, which implies that each of the DoFs $Z_{\text{gen}}$, *τ*, and *β* is a figure of merit; see Figure 1C. At $T_{h}=900\;\text{K}$ and $T_{c}=300\;\text{K}$, the $\eta_{\text{max}}$ for arbitrary linear and quadratic curves varies up to 40% compared to the CPM where τ=β=0; see Figures 3A and 3B. Although $\eta_{\text{max}}$ is roughly proportional to $Z_{\text{gen}}$, the values are dispersed around the CPM efficiency curve. This dispersion shows that the single-parameter generalizations of *zT* fail for some materials and that a multiparameter theory is needed for efficiency prediction. When $Z_{\text{gen}}T_{\text{mid}}$ is constant, $\eta_{\text{max}}$ is proportional to *τ* and *β*, depending on the shape of the thermoelectric property curves. A decreasing Seebeck coefficient *α* with respect to *T* gives a positive *τ*, while an increasing *α* gives a negative *τ*; see Figure 3C. Similarly, the temperature variation of the product of *ρ* and *κ* affects *β*; see Figure 3D.

The efficiency formula in Equation 15 is also applicable to segmented- and graded-material devices with contact resistance. This is because the computation of $Z_{\text{gen}}$, *τ*, and *β* in Equations 9 and 14 is based on the integral formulation of the temperature distribution in Equation 7. The derivative of *α* in the Thomson heat can be replaced by the Peltier and Seebeck effects using integration by parts Tdα=d(αT)−αdT; see STAR Methods (efficiency computation for segmented legs with contact) for details. Then, the choice of α(x), ρ(x), and κ(x) for such general cases is straightforward.

#### Generalized maximum efficiency formula $\eta_{\text{max}}^{\text{gen}}$

Although the DoFs depend on *γ*, the dependency is negligible near the $\gamma_{\text{max}}$ where the maximum efficiency occurs because the temperature distribution hardly changes near the $\gamma_{\text{max}}$ in most thermoelectric materials. Hence, we may assume that the DoFs in $\eta \left(Z_{\text{gen}},\tau ,\beta |\gamma \right)$ are fixed, independent values and maximize *η* only for *γ* to find an approximate maximum efficiency. In this way, we have the simple approximate formula of maximum thermoelectric efficiency in Equations 2 with 3 when *γ* is near $\gamma_{\text{max}}^{\text{gen}}$ given as (see STAR Methods [thermoelectric efficiency has three degrees of freedom] for details)

(Equation 16) $$\begin{array}{l} \eta_{\text{max}}^{\text{gen}}:=\eta \left(Z_{\text{gen}},\tau ,\beta |\gamma =\gamma_{\text{max}}^{\text{gen}}\right)=\frac{\Delta T}{T_{h}^{\text{′}}}\frac{\sqrt{1+Z_{\text{gen}}T_{\text{mid}}^{\text{′}}}-1}{\sqrt{1+Z_{\text{gen}}T_{\text{mid}}^{\text{′}}}+\frac{T_{c}^{\text{′}}}{T_{h}^{\text{′}}}},\\ \gamma_{\text{max}}^{\text{gen}}:=\sqrt{1+Z_{\text{gen}}T_{\text{mid}}^{\text{′}}}. \end{array}$$

The generalized maximum efficiency formula $\eta_{\text{max}}^{\text{gen}}\left(Z_{\text{gen}},\tau ,\beta \right)$ is highly accurate such that the difference between the exact numerical efficiency and $\eta_{\text{max}}^{\text{gen}}$ is negligibly small; see STAR Methods (maximum efficiency prediction using ηmaxgen).

### Material and device design using DoFs

#### Material design using DoFs

Our general efficiency theory can explain the high zT but low *η* examples that single-parameter theories could not explain. For the two virtual materials *mat1* and *mat2* in Ryu et al. (2020), the zT curve of *mat2* is higher than that of *mat1* over the whole working temperature range from 300K to 900K, but the efficiency of *mat2* is lower than that of *mat1*; see Figure 3A. A single parameter averaging the zT curve cannot explain this. Our first parameter is not an exception; the $Z_{\text{gen}}$ of *mat2*$=0.0428\;{\text{K}}^{-1}$ is higher than the $Z_{\text{gen}}$ of *mat1*$=0.0317\;{\text{K}}^{-1}$. However, our second parameter *τ* can correct this reversed relation; the *τ* of *mat2* =−0.107 is lower than the *τ* of *mat1* =0. This example shows that a material benefit from a higher *τ* even with a smaller $Z_{\text{gen}}$.

**Figure 4:**
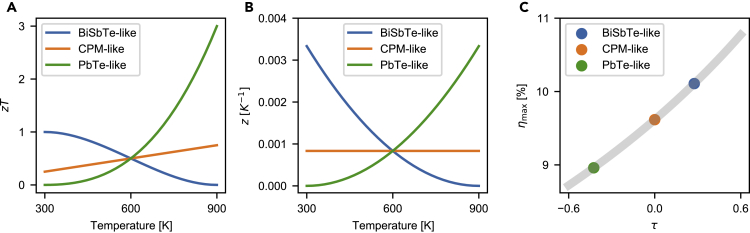
Efficiency and temperature dependency (A) zT, (B) z, and (C) $\eta_{\text{max}}$ and *τ* for three virtual materials that represent BiSbTe-like, CPM-like, and PbTe-like materials. The *α* of the materials is linear while the *ρ* and *κ* are constant. When $T_{c}=300\;\text{K}$ and $T_{h}=900\;\text{K}$, the materials have the same $\bar{\alpha }$ and $Z_{\text{gen}}$. The highest efficiency is found in the BiSbTe-like material due to the positive *τ*.

Next, a more practical example showing the power of *τ* is presented. In Figure 4, three sets of virtual material properties are given, representing BiSbTe-like, CPM-like, and PbTe-like materials. For all three materials, only *α* depends on *T*, and *ρ* and *κ* are constant for working temperatures from $T_{c}=300\;\text{K}$ to $T_{h}=900\;\text{K}$. $\bar{\alpha }$ and $Z_{\text{gen}}T_{\text{mid}}$ are the same for the three materials. The peak zT of the PbTe-like material is significantly higher than that of the other materials (Figure 4A), while the *z* values are comparable to each other (Figure 4B). However, the efficiency of the BiSbTe-like material is higher than that of the other materials as it has a higher *τ* (Figure 4C). The main reason that the BiSbTe-like material has a higher *τ* is that the slope of its Seebeck coefficient is negative. We will see $\tau \approx -\frac{1}{6}\frac{\alpha_{h}-\alpha_{c}}{\int \alpha dT/\text{Δ}T}$ in the following subsection.

**Figure 5:**
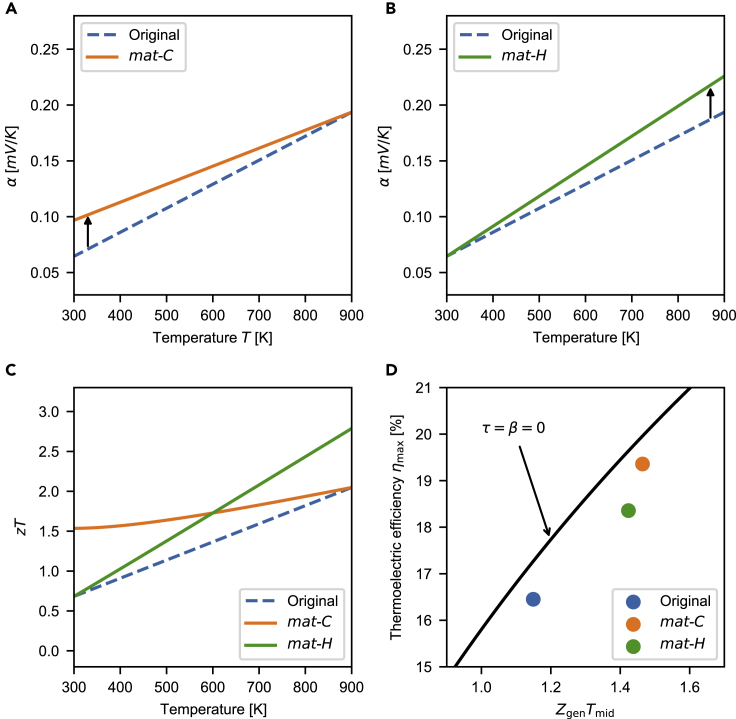
Efficiency enhancement in homogeneous materials (A) Seebeck coefficient curves of *mat-C* with lifted $\alpha \left(T_{c}\right)$ compared to the original curve. (B) Seebeck coefficient curves of *mat-H* with lifted $\alpha \left(T_{h}\right)$ compared to the original curve. (C) zT curves for *mat-C*, *mat-H*, and the original material. Here, the original material has linear α(T), quadratic $\rho \left(T\right)=CT^{2}$, and constant *κ*. (D) $\eta_{\text{max}}$ for *mat-C*, *mat-H*, and the original material, along with the solid line for τ=β=0 (CPM case).

The examples suggest a novel material design strategy. To enhance the efficiency, one needs to increase one of the DoFs. Increasing $Z_{\text{gen}}$ is close to the traditional approach. Increasing *β* may not have a significant impact on the efficiency. However, increasing *τ* is a novel and practical approach. One may increase *τ* by decreasing the slope of α(T). Hence, increasing the cold-side Seebeck coefficient is more advantageous than increasing the hot-side Seebeck coefficient, as the former increases both $Z_{\text{gen}}$ and *τ*; see Figure 5.

#### One-shot approximations of DoFs

Computing the exact DoFs requires the exact temperature distribution T(x), hence computing the efficiency via the *exact* DoFs is not practical because T(x) can give the efficiency directly. Here, we present two *approximations* of the DoFs that do not require the exact T(x): one is highly accurate and the other is very simple. Using the approximated DoFs, the efficiency can be rapidly and accurately estimated.

To derive an approximation of the DoFs, we assume that the temperature distribution inside the leg is $T^{\left(0\right)}\left(x\right)$, which is the temperature distribution for the J=0 case (i.e., $-\kappa \frac{dT}{dx}=\text{const}.$); the superscript (0) indicates that we use the open-circuit J=0 case. By evaluating Equations 9 and 14 with $T=T^{\left(0\right)}$, we can find the *one-shot* approximations of the DoFs denoted by $Z_{\text{gen}}^{\left(0\right)}$, $\tau^{\left(0\right)}$ and $\beta^{\left(0\right)}$. $Z_{\text{gen}}^{\left(0\right)}$ can be explicitly written as

(Equation 17) $$Z_{\text{gen}}^{\left(0\right)}=\frac{{\left(\int \alpha \;dT\right)}^{2}}{\Delta T\int \rho \kappa dT},$$

which is the same as the effective zT of Ioffe and Borrego (Ioffe (1957); Borrego Larralde (1961)). We can further simplify $\tau^{\left(0\right)}$ and $\beta^{\left(0\right)}$ by imposing additional assumptions on *α*, *ρ*, and *κ*. Assuming α(T) and ρ(T)κ(T) are linear with respect to *T*, we have explicit approximation formulas of *τ* and *β*:

(Equation 18) $$\begin{array}{l} \tau_{\text{lin}}^{\left(0\right)}:=-\frac{1}{6}\frac{\alpha_{h}-\alpha_{c}}{\int \alpha \;dT/\text{Δ}T},\\ \beta_{\text{lin}}^{\left(0\right)}:=\frac{1}{6}\frac{\rho_{h}\kappa_{h}-\rho_{c}\kappa_{c}}{\int \rho \kappa \;dT/\text{Δ}T}. \end{array}$$

Here, the subscript “lin” emphasizes the linearity of the material properties. The relation between a positive *τ* and a negative slope of α(T) is clearly shown in $\tau_{\text{lin}}^{\left(0\right)}$. For a detailed derivation, see STAR Methods (One-shot approximation Zgen(0), τlin(0), and βlin(0)).

**Figure 6:**
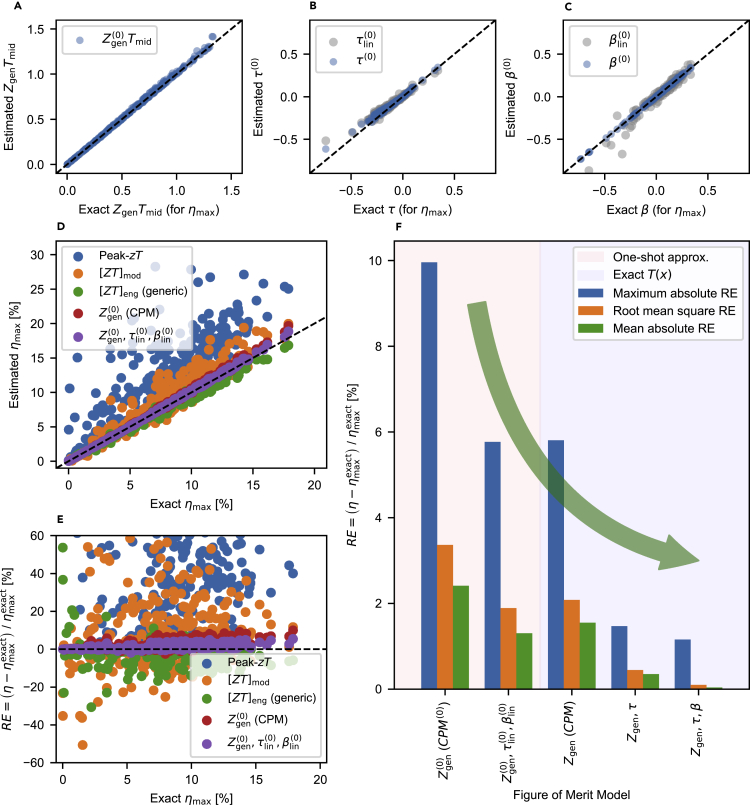
Thermoelectric figures of merit and $\eta_{\text{max}}$ for 277 published materials (A–C) (A), (B), (C) Comparison of the one-shot approximations of $Z_{\text{gen}}^{\left(0\right)}$, $\tau_{\text{lin}}^{\left(0\right)}$, $\beta_{\text{lin}}^{\left(0\right)}$ and the exact $Z_{\text{gen}}$, *τ*, and *β* when the maximum efficiency is attained (also see Table S5). Efficiency estimation methods using the CPM formula with peak zT ($\eta_{\text{max}}^{\text{cpm}}\left(\text{peak}-\text{zT}\right)$), with ${\left[ZT\right]}_{\text{mod}}$ ($\eta_{\text{max}}^{\text{cpm}}\left({\left[ZT\right]}_{\text{mod}}\right)$), and with $Z_{\text{gen}}^{\left(0\right)}$ ($\eta_{\text{max}}^{\text{cpm}}\left(Z_{\text{gen}}^{\left(0\right)}T_{\text{mid}}\right)$), Kim et al.’s generic maximum efficiency formula including ${\left[ZT\right]}_{\text{eng}}$ and the Thomson heat (Kim et al., 2015a, Equation [8]), and $\eta_{\text{max}}^{\text{gen}}$ with one-shot DoFs are tested for 277 published materials under the available temperature range: see STAR Methods (277 published thermoelectric property data, maximum efficiency prediction using ηmaxgen), Tables S3, S4, and S6, and Figure S3 (D) Comparison of the estimated $\eta_{\text{max}}$ with the exact numerical (E) Relative errors between the efficiency estimation method results and the exact numerical $\eta_{\text{max}}$. (F) Comparison of various figure of merit models under the one-shot formula and exact T(x) distribution. With the three thermoelectric DoFs, the estimated maximum efficiency becomes highly accurate. As the efficiency predictions based on $Z_{\text{gen}}^{\left(0\right)}$ under zero *J* (or $Z_{\text{gen}}$ under exact *J*) are done using the general maximum efficiency formula with τ=β=0, we can call them ${\text{CPM}}^{\left(0\right)}$ (or CPM).

The maximum efficiency can be almost exactly estimated by $\eta_{\text{max}}^{\text{gen}}\left(Z_{\text{gen}}^{\left(0\right)},\tau^{\left(0\right)},\beta^{\left(0\right)}\right)$ with the explicit formula of $\eta_{\text{max}}^{\text{gen}}$ given in Equation 2. Figures 6A–6C shows that the difference between the exact DoFs and the one-shot approximations $Z_{\text{gen}}^{\left(0\right)},\tau^{\left(0\right)},\beta^{\left(0\right)}$ is negligible at the maximum efficiency of 277 published materials. The information on the gathered material properties and the publications are given in STAR Methods (277 published thermoelectric property data) and Tables S1 and S9.

The simple formulas of $\tau_{\text{lin}}^{\left(0\right)}$ and $\beta_{\text{lin}}^{\left(0\right)}$ are also capable of accurately estimating the maximum efficiency $\eta_{\text{max}}$. Figures 6D and 6E show that $\eta_{\text{max}}^{\text{gen}}\left(Z_{\text{gen}}^{\left(0\right)},\tau_{\text{lin}}^{\left(0\right)},\beta_{\text{lin}}^{\left(0\right)}\right)$ accurately estimates $\eta_{\text{max}}$. Its root mean squared relative error (RMSRE) is only 1.9% for 277 published materials; see STAR Methods (maximum efficiency prediction using ηmaxgen), Figure S3; Tables S1–S6 and S9). Even using the $\eta_{\text{max}}^{\text{gen}}\left(Z_{\text{gen}}^{\left(0\right)},0,0\right)$ ignoring *τ* and *β* can be a good estimation, showing why the CPM can work well for some cases while failing for other cases, as previously reported (Sherman et al. (1960); Ponnusamy et al. (2020a, 2020b)). In the same figure, our approximation method is compared to other figure of merit models: the peak zT, the ${\left[ZT\right]}_{\text{mod}}$ of Min et al. (Min et al. (2004)) and the engineering ${\left[ZT\right]}_{\text{eng}}$ generic formula of Kim et al. (Kim et al. (2015a)). The peak zT highly overestimates $\eta_{\text{max}}$. The modified ${\left[ZT\right]}_{\text{mod}}$ has an RMSRE of 24.4%. The engineering ${\left[ZT\right]}_{\text{eng}}$ has an RMSRE of 7.2%.

High-throughput screening of candidate materials in a target temperature range is possible via the simple formula $\eta_{\text{max}}^{\text{gen}}\left(Z_{\text{gen}}^{\left(0\right)},\tau_{\text{lin}}^{\left(0\right)},\beta_{\text{lin}}^{\left(0\right)}\right)$. To quantify this capability, the Kendall rank correlation coefficient (see, e.g. Hogg et al., 2005, Section 10.8.1), measuring the monotonicity between approximate formulas and the exact efficiency is estimated; see STAR Methods (Kendall rank correlation coefficients of maximum efficiency estimation methods), Figures 6D and 6E, and Table S7. If the Kendall rank correlation coefficient is close to 1, then the approximate formula well preserves the original rank on the order of the exact efficiency. For ΔT=100K, our formula has the highest correlation coefficient of 0.9993, while ${\left[ZT\right]}_{\text{eng}}$ and ${\left[ZT\right]}_{\text{mod}}$ have similar but lower correlation coefficients of 0.9956 and 0.9722, respectively. For a larger temperature range of ΔT=600K, the discrepancy in the coefficients becomes larger such that our formula has a correlation coefficient of 0.9724, ${\left[ZT\right]}_{\text{eng}}$ has 0.9060, and ${\left[ZT\right]}_{\text{mod}}$ has 0.7657.

#### DoFs of segmented legs and modules

The one-shot approximations $Z_{\text{gen}}^{\left(0\right)}$, $\tau_{\text{lin}}^{\left(0\right)}$, and $\beta_{\text{lin}}^{\left(0\right)}$ of the DoFs in Equation 4 can be generalized for segmented legs. Suppose, a segmented leg is composed of *N* materials with the properties $\alpha_{i}\left(T\right)$, $\rho_{i}\left(T\right)$, and $\kappa_{i}\left(T\right)$ in the spatial interval $\left[L_{i-1},L_{i}\right]$ for i=1,⋯,N. Here, $L_{0}=0$ and $L_{N}=L$. As $T^{\left(0\right)}\left(x\right)$ is strictly decreasing with respect to *x*, there is a unique $T_{i}$ such that $T^{\left(0\right)}\left(L_{i}\right)=T_{i}$ for i=1,⋯,N. Then, ${\bar{\alpha }}^{\left(0\right)}$ and ${\left(\bar{\rho }\bar{\kappa }\right)}^{\left(0\right)}$ in Equation 4 can be generalized for segmented legs as

(Equation 19) $$\begin{array}{l} {\bar{\alpha }}^{\left(0\right)}:=\sum_{i=1}^{N}\int_{T_{i-1}}^{T_{i}}\alpha_{i}\left(T\right)\;dT,\\ {\left(\bar{\rho }\bar{\kappa }\right)}^{\left(0\right)}:=\sum_{i=1}^{N}\int_{T_{i-1}}^{T_{i}}\rho_{i}\left(T\right)\cdot \kappa_{i}\left(T\right)\;dT. \end{array}$$

Here, *N* is the number of segmentes in a leg. Plugging this definition into Equation 4, $Z_{\text{gen}}^{\left(0\right)}$, $\tau_{\text{lin}}^{\left(0\right)}$, and $\beta_{\text{lin}}^{\left(0\right)}$ are defined. For modules consisting of *p*- and *n*-leg pairs, the DoFs can be naturally generalized by summing the power and heat currents; see STAR Methods (module parameters).

The accuracy of the one-shot approximations for segmented legs is tested. For a two-stage segmented leg composed of SnSe and BiSbTe at ΔT=670K, the relative error in the efficiency is less than 5% near γ=1; see STAR Methods (accuracy of the one-shot approximation Zgen(0), τlin(0), and βlin(0) for segmented leg) and Figure S4.

In high-performance materials, a higher $Z_{\text{gen}}^{\left(0\right)}$ usually implies higher efficiency even for segmented materials. Hence, high-efficiency materials or modules can be quickly screened out by using $Z_{\text{gen}}^{\left(0\right)}$ only. As an example, the efficiencies of 5-stage segmented legs composed of 18 candidate materials (STAR Methods (18 selected high-zT materials, efficiency computation for segmented legs with contact) and Figure S5) are computed. Of nearly two million combinations of the segmented legs ($18^{5}=1,889,568$), a top 1% high-$Z_{\text{gen}}^{\left(0\right)}$ device is also a top 1% high-efficiency device with 82% probability; see STAR Methods (estimation of efficiency rank using Zgen(0)) and Table S8.

#### Possible impact of DoFs

**Figure 7:**
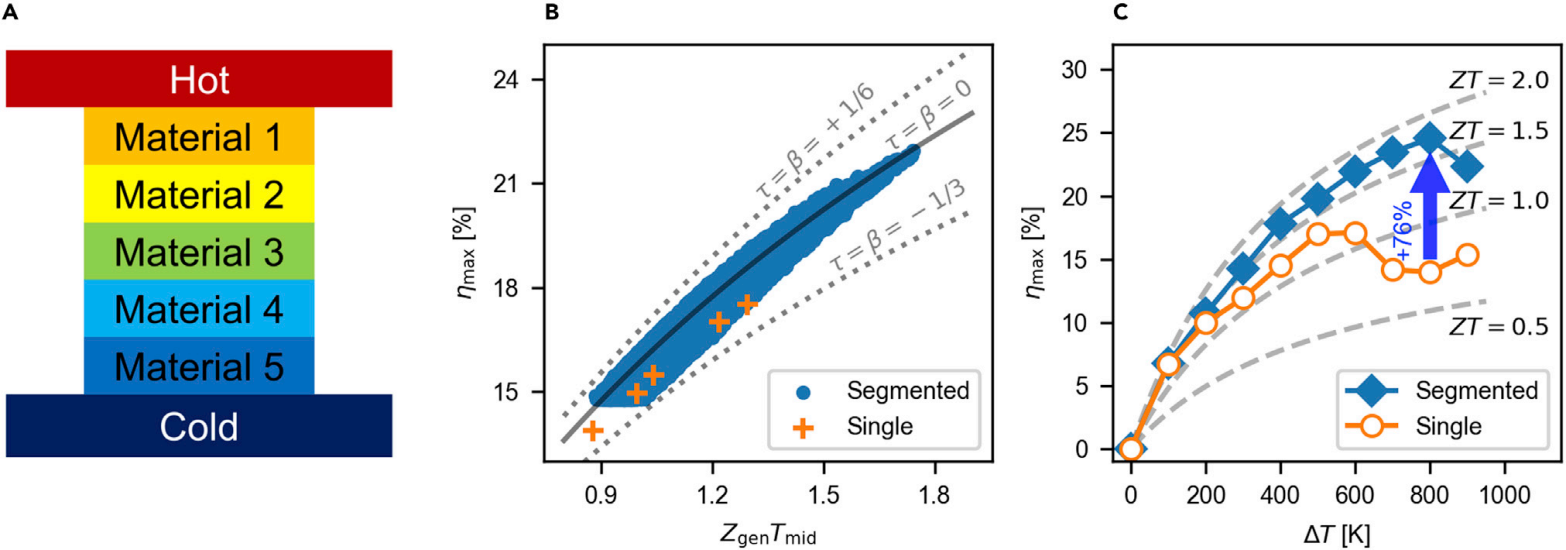
Efficiency enhancement in segmented device (A) The thermoelectric segmented leg is composed of 5 segments with different material properties. (B) Maximum efficiencies for single-material legs (orange cross) and segmented legs (blue circle) for $T_{c}=300\;\text{K}$ and ΔT=600K. For the segmented legs, we consider $18^{5}=1,889,568$ configurations up to 5-stage segmentation consisting of 18 candidate materials; for material and leg information, see STAR Methods (18 selected high zT materials, efficiency computation for segmented legs with contact) and Figure S5. Here, only the top 100,000 configurations are shown. Note that the segmentation can control the DoFs (Figures S6–S9). Additionally, not only does $Z_{\text{gen}}$ affect the efficiency, but also, *τ* and *β* affect the efficiency. (C) Maximum possible efficiencies among single-material legs (orange open circle) and segmented legs (blue filled diamond) for $T_{c}=300\;\text{K}$ and given ΔT. The leg segmentation enhances the maximum efficiency by up to 76% at ΔT=800K.

We have seen that the modulation of the DoFs in materials and devices results in enhanced efficiency. To quantify the impact of the modulation, we explore the efficiency space of 5-stage segmented legs using 18 candidate materials; see Figure 7, STAR Methods (18 selected high-zT materials, efficiency computation for segmented legs with contact, impact of gradient parameters τ and β in segmented legs), and Figures S5–S9. For the moderate efficiency segments having $Z_{\text{gen}}T_{\text{mid}}=1.2$, the efficiency varies up to 10% depending on *τ* and *β*. Further, we achieve a theoretical maximum efficiency $\eta_{\text{max}}$ of 21.9% at ΔT=600K. This is a 28% enhancement compared to single legs. At ΔT=800K, we can expect a higher efficiency enhancement of 76%.

As another example, a design of high-efficiency graded legs using Bi_2_Te_3_ is given in STAR Methods (calculation of thermoelectric properties of Bi2Te3, design of high-efficiency graded legs using Bi2Te3), and Figures S10 and S11. By finding the optimal carrier concentration of each segmented region, a 14% enhancement in the maximum efficiency is expected.

#### Electrical and thermal engineering under contact resistance

As our DoFs determine the input and output heat currents of thermoelectric modules (see Equation 10), they can be used to describe a larger system where contact resistance is important. Here, we present an *algebraic equation* that predicts the efficiency under given thermal and electrical contact resistances.

Owing to the temperature drop at the hot- and cold-side contacts, the effective temperatures for a module are changed from $T_{h}$ and $T_{c}$ to ${\hat{T}}_{h}$ and ${\hat{T}}_{c}$, respectively. Once we find ${\hat{T}}_{h}$ and ${\hat{T}}_{c}$, we can calculate the efficiency as before. By defining new variables $h_{1}:=\frac{\tau }{\text{Δ}T}$ and $h_{2}:=\frac{\beta }{\text{Δ}T}$, we obtain three parameters $Z_{\text{gen}}$, $h_{1}$, and $h_{2}$, which are nearly constant with respect to the electric current *I*. When the leg is contacting an interface with a thermal conductivity of $K_{\text{C}}$, the heat currents at the contacting interface ${\hat{Q}}_{h}$ and ${\hat{Q}}_{c}$ satisfy

(Equation 20) $$\begin{array}{l} {\hat{Q}}_{h}=K\left({\hat{T}}_{h}-{\hat{T}}_{c}\right)+I\bar{\alpha }\left[{\hat{T}}_{h}-h_{1}{\left({\hat{T}}_{h}-{\hat{T}}_{c}\right)}^{2}\right]-\frac{1}{2}I^{2}R\left(1+h_{2}\left({\hat{T}}_{h}-{\hat{T}}_{c}\right)\right),\\ {\hat{Q}}_{c}=K\left({\hat{T}}_{h}-{\hat{T}}_{c}\right)+I\bar{\alpha }\left[{\hat{T}}_{c}-h_{1}{\left({\hat{T}}_{h}-{\hat{T}}_{c}\right)}^{2}\right]+\frac{1}{2}I^{2}R\left(1-h_{2}\left({\hat{T}}_{h}-{\hat{T}}_{c}\right)\right). \end{array}$$

Additionally, by definition of the thermal contact resistance, we have

(Equation 21) $$\begin{array}{l} {\hat{Q}}_{h}=Q_{h}=K_{\text{C}}\left(T_{h}-{\hat{T}}_{h}\right),\\ {\hat{Q}}_{c}=Q_{c}=K_{\text{C}}\left({\hat{T}}_{c}-T_{c}\right). \end{array}$$

Equating Equations 20 and 21 gives a system of quadratic equations for the unknown variables ${\hat{T}}_{h}$ and ${\hat{T}}_{c}$. By solving the system of quadratic equations, we can find ${\hat{T}}_{h}$ and ${\hat{T}}_{c}$, and then ${\hat{Q}}_{h}$ and ${\hat{Q}}_{c}$. Hence, the power $\hat{P}={\hat{Q}}_{h}-{\hat{Q}}_{c}$ and efficiency $\hat{\eta }=\hat{P}/{\hat{Q}}_{h}$ are fully determined using algebraic Equation 20. This approach is validated empirically for computations of effective temperatures and thermoelectric performances (power, heat, and efficiency); see Figures S12 and S13.

The formula can be further extended to the case of electrical contact resistance by adding resistive segments at the interfaces between materials or at the ends of legs.

### Conclusions

Three DoFs in thermoelectrics, $Z_{\text{gen}}$, *τ*, and *β*, determine the thermoelectric conversion efficiency, and they can be easily estimated from material properties. Each DoF is a figure of merit, so improving one of them increases the efficiency. In particular, increasing *τ* is a novel and practical way to increase the efficiency, providing a new route for material discovery and device design, beyond zT.

### Limitation of the study

This paper is based on theoretical formulation and computational validation using thermoelectric property data. Efficiencies reported in this paper are not experimentally measured but computed numerically. For efficiency calculations, we assumed that there is a unique temperature-distribution solution for a thermoelectric equation.

## STAR★Methods

### Key resources table

**Table:** 

REAGENT or RESOURCE	SOURCE	IDENTIFIER
**Software and algorithms**		

Python 3.6.10	Python software foundation	http://www.python.org/ RRID:SCR_008394
NumPy 1.18.5	NumPy project and community	http://www.numpy.org/ RRID:SCR_008633
SciPy 1.5.0	SciPy developers	http://www.scipy.org/ RRID:SCR_008058

### Resource availability

#### Lead contact

Further information and requests for resources should be directed to and will be fulfilled by the lead contact, Byungki Ryu (byungkiryu@keri.re.kr).

#### Materials availability

This study did not generate new unique reagents.

### Additional resources

There are two related preprint versions of this paper: arXiv:1810.11148 and arXiv:1910.11132 by same authors.

### Method details

#### Efficiency computation

Numerical maximum efficiencies of ideal one-dimensional thermoelectric devices are computed for given thermoelectric property curve sets, without thermal loss by radiation or air convection. The thermoelectric property curves are linearly interpolated at intermediate temperature and extrapolated as constant values at the endpoint temperatures. The exact temperature distribution T(x) of the steady state is determined by solving the *integral* Equation 7.

The thermoelectric performances of a thermoelectric leg with the length *L* and cross sectional area *A* are calculated as a function of current density *J* given as $\eta \left(J\right)=\frac{P/A}{Q_{h}/A}=\frac{J\left(\int_{c}^{h}\alpha dT-J\int_{0}^{L}\rho dx\right)}{-\kappa_{h}\nabla T_{h}+J\alpha_{h}T_{h}}$, where the *P* and $Q_{h}$ are the power delivered to the outside and the hot-side heat current respectively. The maximum numerical efficiency $\eta_{\text{max}}$, which satisfies the relation $\eta \left(J\right)\leq \eta_{\text{max}}$ for any *J*, is obtained by Brent-Dekker optimization method.

#### 277 published thermoelectric property data

In this work, we constructed a dataset of thermoelectric properties (TEPs) of 277 materials gathered from 264 literatures to test our theory: for the references see the below and Table S9. The TEPs were digitized using the Plot Digitizer (http://plotdigitizer.sourceforge.net/). The dataset consists of Seebeck coefficient *α*, electrical resistivity *ρ*, and thermal conductivity *κ* at measured temperature *T*. For the numerical computation of efficiency, we use the available temperature ranges of the given material: the $T_{c}$ is defined as the maximum of the lowest measured temperautre and $T_{h}$ is defined as the minimum of the highest measured temperature for given materials.

##### 264 literatures for 277 materials

Biswas et al. (2011, 2012); Fu et al. (2015a); Gelbstein et al. (2013); He et al. (2015a); Heremans et al. (2008); Hsu et al. (2004); Hu et al. (2014, 2016); Kim et al. (2015c); Lin et al. (2016); Liu et al. (2011, 2012b); Pan and Li (2016); Pei et al. (2011g, 2011c); Poudel et al. (2008); Rhyee et al. (2009); Wang et al. (2014a); Zhao et al. (2012a, 2012b, 2014, 2015); Cui et al. (2007, 2008); Eum et al. (2015); Fan et al. (2010); Han et al. (2013); Hsu et al., 2014; Zheng et al. (2014); Hu et al. (2015); Hwang et al. (2013); Ko et al. (2013); Zhang et al. (2015b); Zhao et al. (2005); Lee et al. (2010, 2013c, 2013a); Yan et al. (2010); Lee et al. (2013b, 2014c, 2014b, 2014a, 2014d); Sumithra et al. (2011); Lukas et al. (2012); Min et al. (2013); Mun et al. (2015); Ovsyannikov et al. (2015); Puneet et al. (2013); Shin et al. (2014); Son et al. (2012, 2013); Soni et al. (2012); Xiao et al. (2014); Tang et al. (2007); Wang et al. (2013b); Wu et al. (2013); Yelgel and Srivastava (2012); Zhang et al. (2012a); Wei et al. (2016); Lan et al. (2012); Yu et al. (2013); Kosuga et al. (2014); Scheele et al. (2011); Ahn et al. (2009, 2010, 2013); Androulakis et al. (2010, 2011); Bali et al. (2013, 2014); Wu et al. (2015a); Dong et al. (2009b); Dow et al. (2010); Falkenbach et al. (2014); Fan et al. (2015); Fang et al. (2013); Jaworski et al. (2013); Jian et al. (2015); Keiber et al. (2013); Kim et al. (2016); Lee et al. (2012, 2014f); Li et al. (2013, 2014); Liu et al. (2013b); Lo et al. (2012); Lu et al. (2013); Pei et al. (2011a, 2011e, 2011f, 2011d, 2012a, 2012b, 2014); Poudeu et al. (2006); Rawat et al. (2013); Wang et al. (2013a, 2014b); Wu et al. (2014c, 2014b); Yamini et al. (2015); Yang et al. (2015); Zebarjadi et al. (2011); Zhang et al. (2012d, 2012c, 2013b, 2015c); Al Rahal Al Orabi et al. (2015); Banik et al. (2015, 2016); Banik and Biswas (2016); Chen et al. (2014, 2016); Leng et al. (2016); Pei et al. (2016b); Tan et al. (2014, 2015b, 2015a); Tang et al. (2016); Wang et al. (2015); Zhang et al. (2013a); Zhou et al. (2014); Guan et al. (2015); Suzuki and Nakamura (2015); Fahrnbauer et al. (2015); Gelbstein et al. (2007a, 2007b, 2010); Hazan et al. (2015); Kusz et al. (2016); Lee et al. (2014e); Schröder et al. (2014b, 2014a); Williams et al. (2015); Wu et al. (2014a); Aikebaier et al. (2010); Chen et al. (2012); Dow et al. (2009); Drymiotis et al. (2013); Du et al. (2014); Guin and Biswas (2015); Han et al. (2012); He et al. (2012); Hong et al. (2014); Liu et al. (2016); Mohanraman et al. (2014); Pei et al. (2011b); Wang et al. (2008a); Wu et al. (2015b); Zhang et al. (2010); Aizawa et al. (2006); Akasaka et al. (2007b, 2007a); Cheng et al. (2016); Duan et al. (2016); Kajikawa et al. (1998); Liu et al. (2015); Luo et al. (2009); Mars et al. (2009); Noda et al. (1992); Tani and Kido (2005, 2007a, 2007b); Yang et al. (2009b); Yin et al. (2016); Zhang et al. (2008b, 2008a, 2015a); Zhao et al. (2009b); Joshi et al. (2008); Tang et al. (2010); Wang et al. (2008b); Ahn et al. (2012); Bhatt et al. (2014); Fu et al. (2015b); Kraemer et al. (2015); Krez et al. (2015); Liu et al. (2007b); Mudryk et al. (2002); Shi et al. (2008); Bai et al. (2009); Bao et al. (2009); Chitroub et al. (2009); Dong et al. (2009a); Duan et al. (2012); Dyck et al. (2002); He et al. (2007, 2008); Laufek et al. (2009); Li et al. (2005); Liang et al. (2014); Liu et al. (2007a); Mallik (2008); Mallik et al. (2013); Mi et al. (2008); Pei et al. (2008); Qiu et al. (2011); Rogl et al. (2010, 2011, 2014, 2015); Sales et al. (1996); Shi et al. (2011); Stiewe et al. (2005); Su et al. (2011); Tang et al. (2001); Xu et al. (2014); Yang et al. (2009a); Zhang et al. (2012b); Zhao et al. (2009a); Zhou et al. (2013); Bali et al. (2016); Ding et al. (2016); Jo et al. (2016); Joo et al. (2016); Li et al. (2016b, 2016a); Liu et al. (2012c); Zhou et al. (2017b, 2017a); Zhang et al. (2017); Xu et al. (2017); Xie et al. (2017); Wang et al. (2016); Seo et al. (2017); Pei et al. (2016a); Park et al. (2016); Moon et al. (2016); Zhu et al. (2007); Choi et al. (1997); Yamanaka et al. (2003b); Yang et al. (2008, 2010); Zhang et al. (2009); Zhou et al. (2008); Sharp (2003); Salvador et al. (2009); Levin et al. (2011); Yamanaka et al. (2003a); Zhao et al. (2008, 2006); Yu et al. (2009); Xiong et al. (2010); Toberer et al. (2008); Chung et al. (2000); Tang et al. (2008); Mi et al. (2007); Liu et al. (2008); Li et al. (2008); Chen et al. (2006); Zhong et al. (2014); Yu et al. (2012); Liu et al. (2013a, 2012a); He et al. (2015b); Gahtori et al. (2015); Day et al. (2014); Ballikaya et al. (2013); Bailey et al. (2016); Li et al. (2017); Liang et al. (2017); Isoda et al. (2007).

As shown in Table S1, the 277 materials in our dataset have various base-material groups: 59 Bi_2_Te_3_-related materials, 55 PbTe-related materials, 40 skutterudite (SKD), 23 Mg_2_Si-based materials, 18 GeTe materials, 14 M_2_Q antifluorite-type chalcogenide materials (where M = Cu, Ag, Au and Q = Te, Se), 12 SnTe-related materials, 11 ABQ_2_-type materials (where A=Group I, B=Bi, Sb, Q=Te, Se), 8 SnSe-related materials, 7 PbSe-related materials, 7 half-Heusler (HH) materials, 6 SiGe-related materials, 3 ${\text{In}}_{4}{\text{Se}}_{3}$-related materials, 3 PbS-related materials, 2 oxide materials, 2 clathrate materials, 1 Zintle materials, and 6 others. Here the base material denotes the representative material, not the exact composition. Also note that for the categorization of the base materials, the doping element is ignored. For example, Bi_2_Te_3_, Sb_2_Te_3_, Bi_2_Se_3_ binary and their ternary alloys are categorized as Bi_2_Te_3_-related materials. The material doping composition is not denoted in the composition of the base material.

#### 18 selected high zT materials

For segmented-leg devices, we consider 18 candidates showing high peak-zT values exceeding 1. The full zT curves of them are shown in Figure S5. Tables S2–S5 contain more information on the materials, including available temperature range, peak zT, numerical efficiency, formula efficiency, and the thermoelectric degrees of freedom.

#### Randomly generated thermoelectric properties

The followings describe the sequential procedure used to generate the TEP curves for Figure 1B.

##### Generation of random numbers

We generate 10,000 TEP curve sets for α(T), ρ(T), and κ(T) using a random variable having a uniform probability distribution. For each *α*, *ρ*, and *κ*, we generate three random numbers $y_{1}$, $y_{2}$ and $y_{3}$. For the α(T) curve, the random numbers are between 0 and 1. For the ρ(T) and κ(T) curves, the random numbers are between 1 and 4. Thus, we generate 9 random numbers for the curve shape.

###### Construction of quadratic curves

For each *α*, *ρ*, and *κ*, we generate a quadratic polynomial connecting $\left(300\;\text{K},y_{1}\right)$, $\left(600\;\text{K},y_{2}\right)$, and $\left(900\;\text{K},y_{3}\right)$. We consider the quadratic polynomial as a thermoelectric property curve from 200 K to 1100 K. Using an additional random number between 0 and 4, we resize the curve. The last random number is used to tune the size of average ZT. Here, we use the effective $Z_{\text{gen}}^{\left(0\right)}T_{\text{mid}}$ for *T* range from 200 K to 1100 K (effective zT or general $Z_{\text{gen}}T_{\text{mid}}$ at zero current). Thus, the generated random TEP curves are normalized by the randomly generated $Z_{\text{gen}}^{\left(0\right)}T_{\text{mid}}$ value betwen 0 and 5.

The original quadratic polynomials $\kappa_{\text{o}}\left(T\right)$ and $\rho_{\text{o}}\left(T\right)$ for *κ* and *ρ* are normalized to obtain the TEP curves κ(T) and ρ(T) by

$$\kappa \left(T\right):=\frac{\kappa_{\text{o}}\left(T\right)}{\frac{1}{\Delta T}\int \kappa_{\text{o}}\left(T\right)dT},\;\rho \left(T\right):=\left(10^{-5}\;\text{Ω}\cdot \text{m}\right)\cdot \frac{\rho_{\text{o}}\left(T\right)\times \int \kappa_{\text{o}}\left(T\right)dT}{\int \rho_{\text{o}}\left(T\right)\;\times \;\kappa_{\text{o}}\left(T\right)\;dT},$$

to be certain that $\frac{1}{\text{Δ}T}\int \kappa \left(T\right)dT=1\;\text{W}/\text{m}$ and $\frac{1}{\text{Δ}T}\int \rho (T)\times \kappa \left(T\right)dT=10^{-5}\;\text{Ω}\cdot \text{W}/\text{K}$. The original quadratic polynomial $\alpha_{\text{o}}\left(T\right)$ is normalized as

$$\alpha \left(T\right):=\frac{\alpha_{\text{o}}\left(T\right)}{\frac{1}{\Delta T}\int \alpha_{\text{o}}\left(T\right)dT}\times \sqrt{\frac{Z_{\text{gen}}^{\left(0\right)}T_{\text{mid}}}{T_{\text{mid}}}\times \left(10^{-5}\;\text{Ω}\cdot \text{W}/\text{K}\right)},$$

to be certain that ${\left(\frac{1}{\Delta T}\int \alpha \left(T\right)dT\right)}^{2}=Z_{\text{gen}}^{\left(0\right)}\times \left(10^{-5}\;\text{Ω}\cdot \text{W}/\text{K}\right)$ and $\frac{{\left(\int \alpha \left(T\right)dT\right)}^{2}}{\text{Δ}T\int \rho \left(T\right)\times \kappa (T)\;dT}T_{\text{mid}}=Z_{\text{gen}}^{\left(0\right)}T_{\text{mid}}$

###### Selection of physically meaningful curves

Among the normalized quadratic curves, we only consider the curves satisfying that zT<20, ρ(T)>0, and κ(T)>0 for temperature range from 200 K to 1100 K. Finally, we obtained 4,725 randomly generated TEP curve sets.

###### Digitization of curves and extrapolation

As our efficiency computation code linearly interpolate digitized TEP values, we digitize the generated curves at T=200 K, 225 K, 250 K, ⋯,1100 K. As a consequence, linearly interpolated TEP curves are used.

Sometimes extrapolation is required. Since we may treat high zT cases, the temperature inside a leg can be higher than the hot-side temperature $T_{h}$ (Chung and Ryu, 2014). In this case we need TEP values at T>1100 K. We use constant extrapolation for undefined TEP values.

The efficiency computation may fail to converge when the TEP curve fluctuates severely. We discard this divergent computation case. As a results, we have ‘4,041’, ‘4,648’, ‘4,725’ computation results for $T_{h}=$ 900 K, 600 K, 400 K, respectively, with $T_{c}=300$ K.

#### Calculation of thermoelectric properties of Bi_2_Te_3_

This section describes the calculation method to obtain the Bi_2_Te_3_ thermoelectric properties used in STAR Methods (design of high efficiency graded legs using Bi2Te3).

The thermoelectric properties are calculated using the density functional theory (DFT) (Hohenberg and Kohn (1964); Kohn and Sham (1965)) combined with the Boltzmann transport equation. For the DFT calculations, we use the generalized gradient approximation (GGA) parameterized by PBE (Perdew, Burke, and Ernzerhof) (Perdew et al. (1996)), and the projector augmented wave (PAW) pseudopotential (Blöchl (1994)); both of them are implemented in the **VASP** code (Kresse and Furthmüller (1996); Kresse and Joubert (1999)). The experimental lattice parameters for Bi_2_Te_3_ are used, while the internal coordinates are fully relaxed. The electronic band structure is calculated using the spin-orbit interaction. The *k*-point mesh of 36×36×36 is used. The electronic transport properties are predicted using the DFT band structure coupled with the Boltzmann transport equation within a rigid band approximation and the constant relaxation time approximation; they are implemented in **BoltzTraP** code (Madsen and Singh (2006); Ryu et al. (2017)). Note that we use the experimental band gap of 0.18 eV. The phonon thermal conductivity is calculated using phono3py code (Togo et al. (2015); Ryu and Oh (2016)). The force constants are obtained from the 240-atom supercell with the two-atom displacements using **VASP** code with the single *k*-point Γ and then the third-order phonon Hamiltonian is constructured. The three phonon scattering rates are calculated using the Fermi's golden rule. We also include the effective boundary scattering of 10 nm in addition to the three-phonon scattering. Then the thermal conductivity is calculated by integrating the conductivity on the phonon *q*-point mesh of 11×11×11.

##### Integral Equations of T(x) and $\frac{dT}{dx}\left(x\right)$

Here we derive the integral Equation 7. For simplicity, we denote the term with Thomson heat and Joule heat in Equation 5 by $f_{T}\left(x\right)$:

(Equation 22) $$f_{T}\left(x\right):=-T\frac{d\alpha }{dT}\frac{dT}{dx}J+\rho J^{2}.$$

Then the Equation 5 is $\frac{d}{dx}\left(\kappa \frac{dT}{dx}\right)+f_{T}=0$. If the solution $T_{\text{sol}}$ of Equations 5, 6, and 26 is known, we may put $\kappa \left(x\right):=\kappa \left(T_{\text{sol}}\left(x\right)\right)$ and $f\left(x\right):=f_{T_{\text{sol}}}\left(x\right)$ to find a *linear* differential equation

(Equation 23) $$\frac{d}{dx}\left(\kappa \left(x\right)\frac{dT}{dx}\right)+f=0.$$

Since this equation is linear, we can find the solution *T* by decomposing it into a homogeneous solution $T_{1}$ and a particular solution $T_{2}$ where $T=T_{1}+T_{2}$; see Figure S1. The $T_{1}$ and $T_{2}$ are solutions of

(Equation 24) $$\frac{d}{dx}\left(\kappa \left(x\right)\frac{dT_{1}}{dx}\right)=0,\;T_{1}\left(0\right)=T_{h},\;T_{1}\left(L\right)=T_{c},$$

(Equation 25) $$\frac{d}{dx}\left(\kappa \left(x\right)\frac{dT_{2}}{dx}\right)+f=0,\;T_{2}\left(0\right)=0,\;T_{2}\left(L\right)=0.$$

To solve the Equation 24, we integrate it over *x* to yield $\kappa \left(x\right)\frac{dT_{1}}{dx}\left(x\right)=C$ for some constant *C*. Dividing both sides by *κ* and integrating from 0 to *x*, we have $T_{1}\left(x\right)-T_{1}\left(0\right)=C\int_{0}^{x}\frac{1}{\kappa \left(x\right)}\;dx$. Imposing the boundary conditions yields $C=-K\frac{T_{h}-T_{c}}{A}$ and

$$T_{1}\left(x\right)=T_{h}-\frac{K\Delta T}{A}\int_{0}^{x}\frac{1}{\kappa \left(s\right)}\;ds.$$

To solve the Equation 25, we integrate it from 0 to *x* to yield $\kappa \left(x\right)\frac{dT_{2}}{dx}\left(x\right)-C=-\int_{0}^{x}f\left(s\right)\;ds=:-F\left(x\right)$ for some constant *C*. Dividing both sides by *κ* and integrating from 0 to *x*, we have $T_{2}\left(x\right)-T_{2}\left(0\right)=-\int_{0}^{x}\frac{F\left(s\right)}{\kappa \left(s\right)}\;ds+C\int_{0}^{x}\frac{1}{\kappa \left(s\right)}\;ds$. Imposing the zero boundary conditions yields

$$T_{2}\left(x\right)=-\int_{0}^{x}\frac{F\left(s\right)}{\kappa \left(s\right)}\;ds+\frac{K\;\delta T}{A}\int_{0}^{x}\frac{1}{\kappa \left(s\right)}\;ds,$$

where $\delta T:=\int_{0}^{L}\frac{F\left(x\right)}{\kappa \left(x\right)}\;dx$ is a scalar quantity.

Summing up, we can check that the solution $T=T_{1}+T_{2}$ of Eqautions 23 and 6, and its gradient can be written as Equation 7.

#### Numerical method for finding temperature solution

This section describes the numerical method to find temperature solution at a given *J* or *γ*.

The exact temperature solution T(x) of the thermoelectric equation for a given *J* or *γ* is determined by solving the *integral* Equation 7 with Dirichlet boundary conditions where the end-point temperatures are $T_{h}$ and $T_{c}$. To find the solution, a fixed-point iteration method is used. The precise procedure is the following:

(1) Choose the linear function satisfying the Dirichlet boundary conditions as the initial guess $T_{0}\left(x\right)$ of the exact temperature distribution.
(2) For a given $T_{n}\left(x\right)$, let $\alpha \left(x\right):=\alpha \left(T_{n}\left(x\right)\right)$, $\rho \left(x\right):=\rho \left(T_{n}\left(x\right)\right)$ and $\kappa \left(x\right):=\kappa \left(T_{n}\left(x\right)\right)$.
(3) Compute $T_{n+1}\left(x\right)$ by evaluating the right-hand side of the first integral equation in 7.
(4) If $T_{n+1}\left(x\right)$ agrees with $T_{n}\left(x\right)$, then the $T_{n+1}\left(x\right)$ is the numerical solution. Otherwise go back to the step (2) with $T_{n+1}\left(x\right)$.

Computations reveal that the $T_{n}$ converges within a few iterations (less than 10 iterations) in our cases.

#### Device parameters

The thermoelectric (TE) power device mentioned in this paper is a uni-leg device composed of a single-material leg or a segmented leg sandwiched by heat source ($T_{h}$) and heat sink ($T_{c}$) at both sides. In such a device, electric current and heat current flow simultaneously across the leg. For simplicity, we assume the steady-state condition. For a *p*-type material (α>0), the electric current and the heat current flow in the same direction from hot to cold side, while the direction of the electric current is reversed in a *n*-type material (α<0).

The most important parameters in a TE device are voltage *V*, electrical resistance *R*, and thermal resistance 1/K, which can describe the electrical and thermal circuits of the TE device. Once these three device parameters are known, we can roughly estimate the thermoelectric performance of the TE device. When there is load resistance $R_{\text{L}}$, there will be electric current $I=\frac{V}{R+R_{\text{L}}}$. When there is no electric current, there will be heat current $Q_{h}=-A\kappa \nabla T=K\text{Δ}T$. When there is non-zero electric current, there will be heat generation by Thomson and Joule heat, and the hot-side heat current will be approximately $Q_{h}\approx K\text{Δ}T+I\frac{V}{\text{Δ}T}T_{h}-\frac{1}{2}I^{2}R$. The approximation becomes exact when there is no temperature dependence in TEPs. As soon as the temperature distribution T(x) inside the device is known, the three parameters V,R,K are easily determined from the TEPs. Note that a leg of the device is equivalent to a series of infinitesimal parts dx, and we can write the induced open-circuit voltage (*V*) as the integration of −α∇T on *x*, and the resistance of the TE leg ($R_{TE}$ and $1/K_{TE}$) as the integration of resistivity *ρ* and 1/κ on *x*; see Figure S2. Also note that the electrical and thermal resistances should be calculated by integration of the corresponding resistivities on *x*, not on *T*.

When the material thermoelectric figure of merit zT is small, the electric current density *J* is so small that the *R* and *K* can be estimated by $R^{\left(0\right)}$ and $K^{\left(0\right)}$ that are the electrical resistance and thermal conductance for zero-current-density case (J=0). Similarly, since the *J* is small, the temperature can be estimated by the zero-current-density solution $T^{\left(0\right)}\left(x\right)$ that is the solution of the heat equation ∇⋅(κ∇T)=0 without thermoelectric heat generation. Here the *κ* is thermal conductivity. The heat flows are nearly the same along the thermoelectric leg so the one-dimensional heat equation suggests $\kappa \frac{dT}{dx}$ is constant. Hence the average thermal conductivity ${\bar{\kappa }}^{\left(0\right)}$ for J=0 satisfies ${\bar{\kappa }}^{\left(0\right)}\frac{\text{Δ}T}{L}=\kappa \frac{dT}{dx}$ so it can be evaluated by integration over *T*: ${\bar{\kappa }}^{\left(0\right)}=\int {\bar{\kappa }}^{\left(0\right)}\frac{1}{L}dx=\frac{1}{\text{Δ}T}\int \kappa \frac{dT}{dx}dx=\langle \kappa \rangle_{T}$ by the change of variable $dx=\frac{\kappa dT}{{\bar{\kappa }}_{0}\frac{\text{Δ}T}{L}}$. Here the $\langle \kappa \rangle_{T}$ denotes the average of the thermal conductivity κ(T) over *T*. Meanwhile, the resistivity under the condition J=0 is calculated as ${\bar{\rho }}^{\left(0\right)}=\frac{1}{L}\int \rho dx=\frac{1}{L}\int \rho \frac{\kappa dT}{{\bar{\kappa }}^{\left(0\right)}\frac{\text{Δ}T}{L}}=\frac{1}{{\bar{\kappa }}^{\left(0\right)}\text{Δ}T}\int \rho \kappa dT=\frac{\langle \rho \kappa \rangle_{T}}{\langle \kappa \rangle_{T}}$. Finally we may rewrite $RK=\bar{\rho }\;\bar{\kappa }\approx {\bar{\rho }}^{\left(0\right)}{\bar{\kappa }}^{\left(0\right)}=\langle \rho \kappa \rangle_{T}$ under a small zT.

The above idea to use the device parameters for J=0 is the main idea to derive the one-shot approximation. Every thermoelectric material at the moment has the peak zT smaller than 3, implying that the above idea can give a good approximation $Z_{\text{gen}}^{\left(0\right)}$ for $Z_{\text{gen}}$; see Equation 4 in the manuscript for its definition. However, under large zT or non-zero *J*, the approximation $Z_{\text{gen}}^{\left(0\right)}$ may cause a non-negligible error.

#### Electric current equation

With given load resistance $R_{\text{L}}$, an equation for the electric current density $J=\sigma \left(E-\alpha \frac{dT}{dx}\right)$ can be found by integrating ρJ along the closed circuit: $\oint \rho J\;dx=\oint E\;dx-\oint \alpha \frac{dT}{dx}\;dx=V$. Hence the electric current *I* satisfies $\left(R+R_{\text{L}}\right)I=V$ and we have

(Equation 26) $$J=\frac{1}{A}\frac{V}{R+R_{\text{L}}}.$$

Note that the $R=\frac{1}{A}\int_{0}^{L}\rho \left(T\left(x\right)\right)\;dx$ depends on *T* so does the *J*.

#### Heat current and two additional DoFs *τ* and *β*

This section derives the *τ* and *β* in Equation 14. Using the $\frac{dT}{dx}$ in the Equation 7, the hot-side heat current can be written as

(Equation 27) $$Q_{h}=AJ_{h}^{Q}=I\alpha_{h}T_{h}-A\kappa_{h}{\left(\frac{dT}{dx}\right)}_{h}=I\alpha_{h}T_{h}+K\left(\Delta T-\delta T\right).$$

Now we decompose δT into two terms having *I* and $I^{2}$. From Equation 22 and $F_{T}\left(x\right)=\int_{0}^{x}f_{T}\left(s\right)\;ds$, the Equation 12 follows. Then by $\delta T=\int_{0}^{L}\frac{F_{T}\left(x\right)}{\kappa \left(x\right)}\;dx$, the Equation 13 follows. For *temperature-independent* material properties, we can easily check that $\delta T^{\left(2\right)}=\frac{1}{2}\frac{R}{K}$ and $\delta T^{\left(1\right)}\equiv 0$ so that the hot-side heat current is

$$\bar{Q_{h}}=K\Delta T+I\bar{\alpha }T_{h}-\frac{1}{2}I^{2}R.$$

Our strategy is to consider the $Q_{h}$ in Equation 27 as a perturbation of $\bar{Q_{h}}$ above. To do so, we replace $\alpha_{h}$ by $\bar{\alpha }$ in Equation 27 and introduce dimensionless perturbation parameters *τ* and *β* of which values become zero for temperature-independent material properties. Precisely we define *τ* and *β* as the Equation 14. Then we can rewrite the $Q_{h}$ in Equaiton 27 by

(Equation 28) $$Q_{h}=K\Delta T+I\bar{\alpha }\left(T_{h}-\tau \Delta T\right)-\frac{1}{2}I^{2}R\left(1+\beta \right).$$

Observing the delivered power $P=I\left(V-IR\right)=I\left(\bar{\alpha }\text{Δ}T-IR\right)$ is equal to $Q_{h}-Q_{c}$, we have the cold-side heat current:

$$Q_{c}=K\Delta T+I\bar{\alpha }\left(T_{c}-\tau \text{Δ}T\right)+\frac{1}{2}I^{2}R\left(1-\beta \right).$$

When the average device parameters are fixed, the $Q_{h}$ in Equation 28 decreases as *τ* or *β* increases while the delivered power *P* is fixed. Hence the efficiency $\eta =\frac{P}{Q_{h}}$ increases as *τ* or *β* increases. This implies each of *τ* and *β* is a figure of merit for efficiency, as well as $Z_{\text{gen}}$ is.

#### Thermoelectric efficiency has three degrees of freedom

This section derives the Equations 15 and 16. Let $\gamma :=\frac{R_{\text{L}}}{R}$. Then the electric current is $I=\frac{\bar{\alpha }\text{Δ}T}{R\left(1+\gamma \right)}$ and the delivered power is $P=I\left(\bar{\alpha }\text{Δ}T-IR\right)=\frac{{\left(\bar{\alpha }\text{Δ}T\right)}^{2}}{R}\frac{\gamma }{{\left(1+\gamma \right)}^{2}}$. Using Equation 28, the efficiency $\eta =\frac{P}{Q_{h}}=\frac{P/\left(K\text{Δ}T\right)}{Q_{h}/\left(K\text{Δ}T\right)}$ can be written as

$$\eta \left(Z_{\text{gen}},\tau ,\beta |T_{h},T_{c},\gamma \right)=\frac{Z_{\text{gen}}\Delta T\frac{\gamma }{{\left(1+\gamma \right)}^{2}}}{1+Z_{\text{gen}}\left(\frac{1}{1+\gamma }\right)\left(T_{h}-\tau \Delta T\right)-\frac{1}{2}Z_{\text{gen}}\Delta T{\left(\frac{1}{1+\gamma }\right)}^{2}\left(1+\beta \right).}$$

Dividing the numerator and denominator of the right-hand side by $Z_{\text{gen}}$ gives the Equation 15. Note that, once we know the three DoFs $Z_{\text{gen}}$, *τ* and *β* for a given *J* or *γ*, the thermoelectric efficiency directly follows from the above equation. This is an exact equation and no approximation is used. Hence the thermoelectric efficiency is a function of the three parameters. In other words, *thermoelectric efficiency has three degrees of freedom*. Hence a single figure of merit cannot describe the exact thermoelectric efficiency. Furthermore we can easily check that the efficiency is monotonically increasing with respect to each of $Z_{\text{gen}}$, *τ* and *β* for fixed $T_{h},T_{c}$ and *γ*.

*Assuming*$Z_{\text{gen}}$, *τ*, *β changes little* near the *γ* at the maximum efficiency, we can estimate the solution *γ* of $\frac{\partial \eta }{\partial \gamma }=0$ and estimate the maximum efficiency. Recall the notations of the effective temperatures $T_{h}^{\text{′}}$, $T_{c}^{\text{′}}$ and $T_{\text{mid}}^{\text{′}}$ in Equation 3. Then the solution of $\frac{\partial \eta }{\partial \gamma }=0$, denoted by $\gamma_{\text{max}}$, is approximately written as

$$\gamma_{\text{max}}\approx \gamma_{\text{max}}^{\text{gen}}:=\sqrt{1+Z_{\text{gen}}T_{\text{mid}}^{\text{′}}},$$

which is the second equation in Equation 16. Then the maximum efficiency is approximated by

$$\eta_{\text{max}}\approx \eta_{\text{max}}^{\text{gen}}:=\eta \left(Z_{\text{gen}},\tau ,\beta |\gamma =\gamma_{\text{max}}^{\text{gen}}\right)=\frac{\Delta T}{T_{h}^{\text{′}}}\frac{\sqrt{1+Z_{\text{gen}}T_{\mathrm{mid}}^{\text{′}}}-1}{\sqrt{1+Z_{\text{gen}}T_{\mathrm{mid}}^{\text{′}}}+\frac{T_{c}^{\text{′}}}{T_{h}^{\text{′}}}},$$

which is the first equation in Eqaution 16. The above formula generalizes the classical maximum efficiency formula for temperature-independent material properties because it has the same form as the classical formula and predicts the exact maximum efficiency accurately; see Figure S4 in supplemental information.

#### One-shot approximation $Z_{\text{gen}}^{\left(0\right)}$, $\tau_{\text{lin}}^{\left(0\right)}$ and $\beta_{\text{lin}}^{\left(0\right)}$

This section derives the one-shot approximation Equations 17 and 18. The idea is to use the temperature distribution for J=0, which is similar to the exact temperature distribution because most devices induce small *J* due to the small zT. Let $T^{\left(0\right)}$ be the temperature distribution for J=0 and define

$$\begin{array}{l} {\bar{\alpha }}^{\left(0\right)}:=\frac{1}{\Delta T}\int_{0}^{L}\left(-\alpha (T^{\left(0\right)}\left(x\right)\right)\frac{dT^{\left(0\right)}}{dx}\left(x\right))\;dx=:\frac{V^{\left(0\right)}}{\Delta T},\\ {\bar{\rho }}^{\left(0\right)}:=\frac{1}{L}\int_{0}^{L}\rho \left(T^{\left(0\right)}\left(x\right)\right)\;dx=:\frac{A}{L}R^{\left(0\right)},\\ \frac{1}{{\bar{\kappa }}^{\left(0\right)}}:=\frac{1}{L}\int_{0}^{L}\frac{1}{\kappa \left(T^{\left(0\right)}\left(x\right)\right)}\;dx=:\frac{A}{L}\frac{1}{K^{\left(0\right)}}. \end{array}$$

From the thermoelectric Equation 5 with J=0, we can check that

(Equation 29) $$-\kappa \left(T^{\left(0\right)}\left(x\right)\right)\frac{dT^{\left(0\right)}}{dx}\left(x\right)={\bar{\kappa }}^{\left(0\right)}\frac{\Delta T}{L}.$$

Hence

$$\int_{T_{c}}^{T_{h}}\rho \left(T\right)\kappa \left(T\right)\;dT=\int_{T_{c}}^{T_{h}}\rho \left(T^{\left(0\right)}\right)\left(-\frac{\Delta T}{L}{\bar{\kappa }}^{\left(0\right)}\right)\frac{dx}{dT^{\left(0\right)}}\;dT^{\left(0\right)}=\frac{\Delta T}{L}\int_{0}^{L}\rho \left(T^{\left(0\right)}\left(x\right)\right)\;{\bar{\kappa }}^{\left(0\right)}\;dx=\Delta T\;{\bar{\rho }}^{\left(0\right)}\;{\bar{\kappa }}^{\left(0\right)}.$$

Replacing *T* with $T^{\left(0\right)}$ in $Z_{\text{gen}}=\frac{{\bar{\alpha }}^{2}}{\bar{\rho }\;\bar{\kappa }}$, we have a one-shot approximation for $Z_{\text{gen}}$:

$$Z_{\text{gen}}\approx \frac{{\bar{\alpha }}^{2}}{{\bar{\rho }}^{\left(0\right)}\;{\bar{\kappa }}^{\left(0\right)}}=\frac{{\left(\int \alpha \;dT\right)}^{2}}{\Delta T\;\int \rho \kappa \;dT}=:Z_{\text{gen}}^{\left(0\right)},$$

which is the Equation 17.

*To approximate τ*, *we assume* the Seebeck coefficient is a linear function of *T*:

$$\alpha \left(T\right)\approx \alpha_{\text{lin}}\left(T\right):=\alpha_{h}+\left(\frac{\alpha_{c}-\alpha_{h}}{T_{c}-T_{h}}\right)\left(T-T_{h}\right).$$

In this way we can observe the effect of the gradient of *α* on *τ* more clearly. Since the *τ* in Eqaution 14 has $K\;\delta T^{\left(1\right)}$ term, we estimate a relevant term:

$$F_{T}^{\left(1\right)}\left(s\right)\approx \int_{0}^{s}\frac{1}{A}T\frac{d\alpha_{\text{lin}}}{dT}\left(T\left(x\right)\right)\frac{dT}{dx}\;dx=\int_{T_{h}}^{T\left(s\right)}\frac{1}{A}T\frac{\alpha_{c}-\alpha_{h}}{T_{c}-T_{h}}\;dT=\frac{1}{2A}\frac{\alpha_{c}-\alpha_{h}}{T_{c}-T_{h}}\left(T{\left(s\right)}^{2}-T_{h}^{2}\right)=:\hat{F^{\left(1\right)}}\left(T\left(s\right)\right).$$

Using$-\kappa \frac{dT}{dx}\approx {\bar{\kappa }}^{\left(0\right)}\frac{\text{Δ}T}{L}$ from Equation 29,

$$\delta T^{\left(1\right)}=\int_{0}^{L}\frac{F_{T}^{\left(1\right)}\left(x\right)}{\kappa \left(x\right)}\;dx\approx -\int_{0}^{L}\frac{\hat{F^{\left(1\right)}}\left(T\left(x\right)\right)}{{\bar{\kappa }}^{\left(0\right)}}\frac{L}{\Delta T}\frac{dT}{dx}\;dx=\frac{1}{{\bar{\kappa }}^{\left(0\right)}}\frac{L}{\Delta T}\int_{T_{c}}^{T_{h}}\hat{F^{\left(1\right)}}\left(T\right)\;dT=\frac{1}{2K^{\left(0\right)}}\frac{1}{\Delta T}\frac{\alpha_{c}-\alpha_{h}}{T_{c}-T_{h}}\frac{1}{3}{\left(\Delta T\right)}^{2}\left(-3T_{h}+\Delta T\right)=\frac{\alpha_{h}-\alpha_{c}}{6K^{\left(0\right)}}\left(-3T_{h}+\Delta T\right)=:\hat{\delta T^{\left(1\right)}}$$

where $K^{\left(0\right)}:=\frac{A}{L}{\bar{\kappa }}^{\left(0\right)}$. Therefore we have an one-shot approximation for *τ*:

$$\tau \approx \frac{1}{\bar{\alpha_{\text{lin}}}\Delta T}\left[\left(\bar{\alpha_{\text{lin}}}-\alpha_{h}\right)T_{h}-K^{\left(0\right)}\;\hat{\delta T^{\left(1\right)}}\right]=-\frac{1}{3}\frac{\alpha_{h}-\alpha_{c}}{\alpha_{h}+\alpha_{c}}=-\frac{1}{6}\frac{\alpha_{h}-\alpha_{c}}{\bar{\alpha }}=:\tau_{\text{lin}}^{\left(0\right)},$$

which is the first formula in Eqaution 18.

*To approximate β*, *we assume* the ρκ is a linear function of *T*:

$$\left(\rho \kappa \right)\left(T\right)\approx {\left(\rho \kappa \right)}_{\text{lin}}\left(T\right):={\left(\rho \kappa \right)}_{h}+\left(\frac{{\left(\rho \kappa \right)}_{c}-{\left(\rho \kappa \right)}_{h}}{T_{c}-T_{h}}\right)\left(T-T_{h}\right).$$

*Using*$-\kappa \frac{dT}{dx}\approx {\bar{\kappa }}^{\left(0\right)}\frac{\text{Δ}T}{L}$ from Eqaution 29, we approximate relevant terms for *β*:

$$F_{T}^{\left(2\right)}\left(s\right)=\int_{0}^{s}\frac{1}{A^{2}}\left(\rho \kappa \right)\left(T\left(x\right)\right)\frac{1}{\kappa \left(x\right)}\;dx\approx \frac{-L}{A^{2}{\bar{\kappa }}^{\left(0\right)}\Delta T}\int_{0}^{s}{\left(\rho \kappa \right)}_{\text{lin}}\left(T\left(x\right)\right)\frac{dT}{dx}\;dx=\frac{-L}{A^{2}{\bar{\kappa }}^{\left(0\right)}\Delta T}\int_{T_{h}}^{T\left(s\right)}{\left(\rho \kappa \right)}_{\text{lin}}\left(T\right)\;dT=\frac{-L}{A^{2}{\bar{\kappa }}^{\left(0\right)}\text{Δ}T}\left[{\left(\rho \kappa \right)}_{h}\left(T\left(s\right)-T_{h}\right)+\frac{1}{2}\frac{{\left(\rho \kappa \right)}_{c}-{\left(\rho \kappa \right)}_{h}}{T_{c}-T_{h}}{\left(T\left(s\right)-T_{h}\right)}^{2}\right]=:\hat{F^{\left(2\right)}}\left(T\left(s\right)\right)$$

so that

$$\delta T^{\left(2\right)}=\int_{0}^{L}\frac{F_{T}^{\left(2\right)}\left(x\right)}{\kappa \left(x\right)}\;dx\approx \int_{0}^{L}\hat{F^{\left(2\right)}}\left(T\left(x\right)\right)\left(-\frac{L}{{\bar{\kappa }}^{\left(0\right)}\Delta T}\right)\frac{dT}{dx}\;dx=\frac{-L}{{\bar{\kappa }}^{\left(0\right)}\Delta T}\int_{T_{h}}^{T_{c}}\hat{F^{\left(2\right)}}\left(T\right)\;dT=\frac{1}{6{\left(K^{\left(0\right)}\right)}^{2}}\left(2{\left(\rho \kappa \right)}_{h}+{\left(\rho \kappa \right)}_{c}\right)=:\hat{\delta T^{\left(2\right)}}.$$

Therefore we have a one-shot approximation for *β*:

$$\beta \approx \frac{2}{\frac{L}{A}{\bar{\rho }}^{\left(0\right)}}K^{\left(0\right)}\;\hat{\delta T^{\left(2\right)}}-1=\frac{1}{3\;{\bar{\rho }}^{\left(0\right)}{\bar{\kappa }}^{\left(0\right)}}\left(2{\left(\rho \kappa \right)}_{h}+{\left(\rho \kappa \right)}_{c}\right)-1\approx \frac{1}{\frac{3}{2}\left({\left(\rho \kappa \right)}_{h}+{\left(\rho \kappa \right)}_{c}\right)}\left(2{\left(\rho \kappa \right)}_{h}+{\left(\rho \kappa \right)}_{c}\right)-1=\frac{1}{3}\frac{{\left(\rho \kappa \right)}_{h}-{\left(\rho \kappa \right)}_{c}}{{\left(\rho \kappa \right)}_{h}+{\left(\rho \kappa \right)}_{c}}=\frac{1}{6}\frac{{\left(\rho \kappa \right)}_{h}-{\left(\rho \kappa \right)}_{c}}{{\bar{\rho }}^{\left(0\right)}{\bar{\kappa }}^{\left(0\right)}}=:\beta_{\text{lin}}^{\left(0\right)},$$

which is the second formula in Equation 18.

#### Maximum efficiency prediction using $\eta_{\text{max}}^{\text{gen}}$

In Figure S4, we observe that the maximum efficiency estimation formula $\eta_{\text{max}}^{\text{gen}}\left(Z_{\text{gen}},\tau ,\beta \right)$ in Equation 16 is highly accurate. In Table S6, various statistics on the relative error of maximum efficiency ($\frac{\eta_{\text{max}}^{\text{gen}}-\eta_{\text{max}}}{\eta_{\text{max}}}$) are given.

If we use the exact $Z_{\text{gen}}$, *τ* and *β*, the standard error (=root mean square of relative errors) of $\eta_{\text{max}}^{\text{gen}}$ is $9.60\times 10^{-4}$.

If we use the $Z_{\text{gen}}^{\left(0\right)}$, $\tau_{\text{lin}}^{\left(0\right)}$ and $\beta_{\text{lin}}^{\left(0\right)}$, the standard error is $1.75\times 10^{-2}$. For the single crystalline SnSe with peak zT of 2.6 (Zhao et al. (2014)), the relative error of the one-shot approximation is found to be only $6.82\times 10^{-3}$. However, when we use the different approximation such as linear T(x) or different averaging scheme for *z*, the error becomes larger than ours due to the non-linearity of *T* for the material (Kim et al. (2015a)).

If we only use the $Z_{\text{gen}}^{\left(0\right)}$ with zero *τ* and *β*, the efficiency is still well predicted with the standard error of $3.37\times 10^{-2}$. But, in some materials, the error is relatively large due to the neglect of the *τ* and *β*. The largest relative error of 10% is found for material of reference-(Wu et al. (2014c)), due to the non-vanishing gradient parameters ($\tau =-0.222\approx \tau^{\left(0\right)}=-0.177\approx \tau_{\text{lin}}^{\left(0\right)}=-0.204$, $\beta =0.2085\approx \beta^{\left(0\right)}=0.228\approx \beta_{\text{lin}}^{\left(0\right)}=0.185$, when $T_{h}=918$ K and $T_{c}=304$ K).

#### Kendall rank correlation coefficients of maximum efficiency estimation methods

This section is related to Section ‘One-Shot Approximations of DoFs’. The core of a figure of merit is that a higher figure of merit has higher efficiency. Hence a figure of merit should have a high coefficient of rank correlation with respect to the exact maximum efficiency. To measure this characteristics of our DoFs, we compute the Kendall rank correlation coefficient $\lambda_{\text{rank}}$ (see, e.g., (Hogg et al., 2005, Section 10.8.1)); it is also called the Kendall’s *τ* but here we keep the notation *τ* for our thermoelectric degree of freedom). Let $\left(\eta_{1}^{\text{esti}},\eta_{1}^{\text{exact}}\right),\cdots ,\left(\eta_{n}^{\text{esti}},\eta_{n}^{\text{exact}}\right)$ be a set of estimated and exact maximum efficiencies of thermoelectric property curve sets, where $\eta_{i}^{\text{esti}}$ is an estimated efficiency from figure of merit model and $\eta_{i}^{\text{exact}}$ is an exact maximum efficiency. Any pair of the efficiency set $\left(\eta_{i}^{\text{esti}},\eta_{i}^{\text{exact}}\right)$ and $\left(\eta_{j}^{\text{esti}},\eta_{j}^{\text{exact}}\right)$ (i<j) are told *concordant* if the order is same for $\left(\eta_{i}^{\text{esti}},\eta_{j}^{\text{esti}}\right)$ and $\left(\eta_{i}^{\text{exact}},\eta_{j}^{\text{exact}}\right)$. Otherwise it is said to be *discordant*. Then, the $\lambda_{\text{rank}}$ is defined as

$$\lambda_{\text{rank}}:=\frac{\left(\text{number of concordant pairs}\right)-\left(\text{number of discordant pairs}\right)}{\text{number of possible observations}}.$$

The Kendall rank correlation coefficients of various maximum efficiency estimation methods for 277 published TEP curves are given in Table S7. Our general efficiency formula is superior to the other models when we use the three DoFs. Even further our one-shot approximation is good as a figure of merit model.

#### Accuracy of the one-shot approximation $Z_{\text{gen}}^{\left(0\right)}$, $\tau_{\text{lin}}^{\left(0\right)}$ and $\beta_{\text{lin}}^{\left(0\right)}$ for segmented leg

The one-shot approximations can be used to predict the performance of *segmented* devices. In Figure S4A, we consider a two-stage segmented leg with no contact resistance. The segmented leg consists of SnSe (Zhao et al. (2014)) for hot side and BiSbTe (Poudel et al. (2008)) for cold side. The exact temperature distribution *T* inside the leg shows a jump of the gradient of *T* at x=0.6 due to the inhomogeneity of the material; see Figure S4B. Despite the nonlinearity of the *T*, the one-shot linear approximation, which does not use the exact *T*, shows high accuracy in prediction of thermoelectric performances; see Figures S4C–S4F. The relative error is high near γ=0, where the reaction term is large due to the large electric current and thereby large Joule heat. For large *γ*, the error is negligible. Near the γ=1, the error is acceptable; the relative error is less than 5%. The one-shot linear approximation predicts the maximum efficiency to be 7.67% while the exact value is 7.53%, whereas the one-shot CPM approximation gives 7.73%.

#### Efficiency computation for segmented legs with contact

In this section we present the algorithms we used to compute the efficiency and DoFs for segmented and gradient legs. As the algorithms are based on our formalism of the *integral* Equation 7, they are applicable to segmented legs under contact resistances, by adding resistance blocks on the leg. It implies that we can also treat heat exchangers by including heat exchanger blocks modeled as segmented blocks with thermal interfaces.

##### Leg segmentation

We consider an one-dimensional *p*-type segmented leg. For the segmented leg, we consider a single leg with 5-stage segmentation consisting of 18 *p*-type candidate materials; the information of the candidate materials is given in Table S2 and Figure S5. As all the segmented parts are assumed to have the equal thickness, there are $18^{5}=1,889,568$ configurations.

###### Algorithm for computing the maximum efficiency

For fast computation, we search for the maximum *η* and the optimal *J* altogether. For this purpose, the ‘numerical method for finding temperature solution’ is modified by adding the following steps:

(2-1) Given a temperature distribution $T_{n}$, compute the DoFs using the definition in Equations 9 and 14. Then estimate the optimal current density *J* using the second equation in Equation 16. If a given structure is segmented, the material properties are position-dependent as well as temperature-dependent but there is no additional difficulty in computation.

(5) Using the converged temperature distribution $T_{n+1}$, compute the maximum efficiency using $\eta_{\text{max}}^{\text{gen}}$. For safety, we compute the efficiency using the heat flux equation and numerical temperature gradient, and check whether the efficiency from DoFs and the efficiency from numerical temperature gradient are the same or not.

(6) Finally, the device structure, equivalent device parameters, power, efficiency, and DoFs are reported.

###### Computation time

In a single core computer, the computation of the maximum efficiency of a segmented leg takes less than 1 s. Thus, for total computation under a given temperature condition, it may take about 525 hours (22 days). Thus, for 9 different temperature conditions, we need approximately 200 days for full computation. Fortunately, we used a high-performance-computing (HPC) system consisting of 500 processors, so the computation took about less than 1 day.

*Treatment of the Jump in*α(x). If the material properties α(T), ρ(T), and κ(T) are finite, non-zero and continuous on *T*, then the temperature solution is not singular, and α(x), ρ(x) and κ(x) are continuous. Since the Thomson and Joule heats are also finite (see $f_{T}\left(x\right)$ in the Equation 22), the temperature gradient $\frac{dT}{dx}\left(x\right)$ is also continuous. This fact is consistent to the fact that the Peltier heat is finite owing to the finite Seebeck coeffient.

In segmented legs, α(x) at position *x* is still finite, resulting in the finite Peltier heat; $Q_{\text{Peltier}}=I\alpha \left(x\right)T\left(x\right)<\infty$. However, the α(x) in segmented legs is *discontinuous* at the junction where two different materials meet, which results in inifinite Thomson heat; recall the $\frac{d\alpha }{dx}\left(x\right)$ term in the Thomson heat. Since the Peltier heat is finite while Thomson heat is infinite, the Thomson heat source should be proportional to a Dirac *δ* measure. This behavior of the Thomson heat at the junction makes a discontinuous $\frac{dT}{dx}$ at the junctions. For the numerical computation of the integration of Thomson heat source term in Equation 22, we use the integration by parts:

(Equation 30) $$\int \frac{d\alpha }{dT}\frac{dT}{dx}TJdx=\int TJd\alpha =\alpha TJ-J\int \alpha dT.$$

Now, the above equation can be numerically computed *without treating* a Dirac *δ* measure.

*Physical Meaning of*Equation 30. Integrate the both sides of the thermoelectric Equation 5. Then we obtain the energy-conserving flux-balance equation written as

$$\left(\kappa \frac{dT}{dx}\right){|}_{x}-\left(\kappa \frac{dT}{dx}\right){|}_{x=0}=\left[\left\{\left(\alpha TJ\right){|}_{x}-\left(\alpha TJ\right){|}_{x=0}\right\}-J\left(\int_{0}^{x}\alpha dT-J\int_{0}^{x}\rho dx\right)\right].$$

Note that this is a relation between the four effects in thermoelectricity: heat conduction, Peltier effect, Seebeck effect, and Joule heat. The integral form is exactly the same as the energy conservation differential equation: $\nabla \cdot J^{Q}=E\cdot J$.

##### Treatment of the interface/contact resistance

The electrical interface resistance at a junction also can be treated as a Dirac *δ* measure, also making a discontinuous $\frac{dT}{dx}$ at the junction. But the temperature is still continuous. On the other hand, if there is a thermal interface resistance at the junction, then *κ* is zero at a junction and the temperature distribution becomes discontinuous. Since the heat flux should be finite to conserve the energy, the *κ* at contact should follow the following relation:

$$K_{\text{contact}}\;\Delta T_{\text{contact}}=\underset{L_{c}\to 0}{\text{lim}}\left(A\kappa_{\text{contact}}\frac{\Delta T_{\text{contact}}}{L_{c}}\right).$$

Here, we solve the T(x) with a finite $L_{c}>0$ to bypass the singularity of the equation. Then we take the limit as $L_{c}\to 0$ when calculating the device performance. As we treat the contact material as a finite-volume materials with finite electrical resistivity and thermal resistivity, the leg with electrical and thermal resistance interface can be describe using the segmented leg geometry with $L_{c}>0$. We find that, when the leg size is about 1mm, the interface/contact thickness $L_{c}=0.1\;\text{mm}$ is sufficient to reach the convergent values for the efficiency and power output.

###### Heat exchanger

As a heat exchanger can be replaced with a finite contact thermal resistance equivalenty, our formalism allows the computation under a heat exchanger. By putting the additional thermal resistance segment on the leg, we can simulate the performance of device consisting of a leg and a heat exchanger.

#### Estimation of efficiency rank using $Z_{\text{gen}}^{\left(0\right)}$

The $Z_{\text{gen}}$ is a figure of merit, so a larger $Z_{\text{gen}}$ usually implies a larger maximum efficiency (but be careful that it is not always because the determination of the maximum efficiency requires the additional two DoFs, *τ* and *β*.) If we rank TE devices in order of $Z_{\text{gen}}$, will we get the correct rank in order of the exact maximum efficiency? To measure such an effect quantitatively, we define the *top-rank-preserving probability* by the ratio of the number of correct top ranks predicted by some estimation parameter, to the total number of top ranks.

To test the top-rank-preserving probability, we consider a 5-stage segmented leg in which each segment has the same length. Using the 18 candidate materials in Table S2 and Figure S5, we computed the maximum thermoelectric efficiency of 5-stage segmented legs for all possible configurations; there are $18^{5}=1,889,568$ device configurations. The total length of the leg is 1mm and the cross sectional area is $1{\text{mm}}^{2}$. The hot- and cold-side temperatures are $T_{h}=900$ K and $T_{c}=300$ K. Table S8 shows the top-rank-preserving probability is high even if we use the simplest estimation $Z_{\text{gen}}^{\left(0\right)}$. With 82% probability, the top 1% rank configurations in order of exact maximum efficiency can be found in the top 1% ranks in order of $Z_{\text{gen}}^{\left(0\right)}$. Hence a fast high-throughput screening is possible by computing the $Z_{\text{gen}}^{\left(0\right)}$ only, without computing the numerical maximum efficiency.

##### Additional information

The best efficiency in the setting of Figure 7 and Table S8 is 21.95% while the one-shot approximation $\eta_{\text{max}}^{\text{gen}}\left(Z_{\text{gen}}^{\left(0\right)},\tau^{\left(0\right)},\beta^{\left(0\right)}\right)$ predicts it would be 22.30%. For top 100,000 configurations, the root mean square error is 0.0415.

#### Impact of gradient parameters *τ* and *β* in segmented legs

We demonstrate that the maximum efficiency can be highly enhanced by modulating the gradient parameters *τ* and *β*, even though the $Z_{\text{gen}}$ is fixed. Figures 7 and S6 clarify the relation between the DoFs and the maximum efficiency. We consider 5-stage leg segmention with 18 materials under ΔT=900K−300K to modulate the DoFs. For a given $Z_{\text{gen}}T_{m}=1.2$, the maximum efficiency ranges from 16.6% to 18.2%. But when $Z_{\text{gen}}T_{m}=1.2$ and τ=β=0, the maximum efficiency is 17.7%. Thus, the *τ* and *β* affects the maximum efficiency by up to $\frac{18.2-16.6}{17.7}=9.1\text{\%}$. The relative size change of 9.1% in efficiency corresponds to the absolute size change of 1.25−1.08=0.17 and relative size change of $\frac{1.25-1.08}{1.20}=14.4\text{\%}$ in ${\left[ZT\right]}_{\text{dev}}$, that is the solution of the traditional efficiency equation, ${\left[ZT\right]}_{\text{dev}}:={\left[ZT\right]}_{\text{Snyder}}={\left(\frac{T_{h}-T_{c}\left(1-\eta_{\text{max}}\right)}{T_{h}\left(1-\eta_{\text{max}}\right)-T_{c}}\right)}^{2}-1$ (Snyder and Snyder (2017)). Thus, *τ* and *β* have a significant impact on thermoelectric efficiency.

In Figure S7, the thermoelectric properties of the best segmented leg are shown with respect to temperature and position. In Figures S8 and S9, the thermoelectric properties of the rank 24493 and 54041 segmented legs having $Z_{\text{gen}}T_{\mathrm{mid}}=1.2$ are shown with respect to temperature and position, respectively. Although they have the same $Z_{\text{gen}}T_{\mathrm{mid}}$, they have different thermoelectric conversion efficiency owing to the different shape of thermoelectric properties. In the rank 24493 segmented leg, the Seebeck coefficient is slightly decreasing with *T* and the peak zT value is smaller than 2. Meanwhile, in the rank 54041 segmented leg, the Seebeck coefficient is increasing with *T* and hence the zT is highly increasing with *T* (zT>2 at the hot side). Owing to the temperature dependency, the rank 24493 has a positive *τ* while the rank 54041 has a negative *τ*. In these segmented legs, the role of *τ* is critical; the positve-*τ* segmented leg has a higher conversion efficiency than the negative-*τ* segmented leg.

#### Module parameters

Our formalism of thermoelectric performances on a single or segmented leg can be easily extended to a *p*- and *n*-leg pair modules. First, we set the module parameters from the single-leg device parameters for a given current *I*. Here, for simplicity, we assume that the $T_{h}$ for the *p*-leg, $T_{h}^{\left(p\right)}$, and the $T_{h}$ for the *n*-leg, $T_{h}^{\left(n\right)}$, are the same: $T_{h}^{\left(p\right)}=T_{h}^{\left(n\right)}=T_{h}$. Similarly we assume that the $T_{c}$ for the *p*-leg, $T_{c}^{\left(p\right)}$, and the $T_{c}$ for the *n*-leg, $T_{c}^{\left(n\right)}$, are the same: $T_{c}^{\left(p\right)}=T_{c}^{\left(n\right)}=T_{c}$.

Let *I* be the electric current flowing through the *p*- and *n*-leg, where the *p*- and *n*-legs are connected serially. The current in the *p*-leg flows from hot to cold side and the current in the *n*-leg flows from cold to hot side. The current flows into the *n*-leg cold side, then flows through *n*-leg from cold to hot side, then flows through the electrode connecting *n*- and *p*-legs, then flows through *p*-leg from hot to cold side, then finally flows out from the module. At the same time, the heat input flows parallelly through *p*- and *n*-leg from hot to cold side. Thereby, the current *I*, total internal electrical resistance $R^{\left(pn\right)}$, total open-circuit voltage $V^{\left(pn\right)}$, the total heat input $Q_{h}^{\left(pn\right)}$ satisfies

$$\begin{array}{l} R^{\left(pn\right)}=R^{\left(p\right)}+R^{\left(n\right)},\\ V^{\left(pn\right)}=V^{\left(p\right)}+V^{\left(n\right)}=\left({\bar{\alpha }}^{\left(p\right)}-{\bar{\alpha }}^{\left(n\right)}\right)\text{Δ}T,\\ Q_{h}^{\left(pn\right)}=Q_{h}^{\left(p\right)}+Q_{h}^{\left(n\right)}. \end{array}$$

Considering the I=0 case for voltage and heat current, we define

$$\begin{array}{l} {\bar{\alpha }}^{\left(pn\right)}:={\bar{\alpha }}^{\left(p\right)}-{\bar{\alpha }}^{\left(n\right)},\\ K^{\left(pn\right)}:=K^{\left(p\right)}+K^{\left(n\right)}. \end{array}$$

Here, the current *I* is connected with the outside load resistance $R_{\text{L}}$ so that

$$I\left(R^{\left(pn\right)}+R_{\text{L}}\right)=V^{\left(pn\right)}.$$

Hence the current is described in terms of the module parameters as

$$I=\frac{V^{\left(pn\right)}}{R^{\left(pn\right)}+R_{\text{L}}}=\frac{\left({\bar{\alpha }}^{\left(p\right)}-{\bar{\alpha }}^{\left(n\right)}\right)\text{Δ}T}{R^{\left(p\right)}+R^{\left(n\right)}+R_{\text{L}}}$$

Meanwhile, the total heat current in the module is given as the sum of the heat currents in each leg:

$$\begin{array}{c} Q_{h}^{\left(p\right)}=K^{\left(p\right)}\text{Δ}T+I{\bar{\alpha }}^{\left(p\right)}\left(T_{h}-\tau^{\left(p\right)}\text{Δ}T\right)-\frac{1}{2}I^{2}R^{\left(p\right)}\left(1+\beta^{\left(p\right)}\right),\\ Q_{h}^{\left(n\right)}=K^{\left(n\right)}\text{Δ}T-I{\bar{\alpha }}^{\left(n\right)}\left(T_{h}-\tau^{\left(n\right)}\text{Δ}T\right)-\frac{1}{2}I^{2}R^{\left(n\right)}\left(1+\beta^{\left(n\right)}\right),\\ Q_{h}^{\left(pn\right)}=Q_{h}^{\left(p\right)}+Q_{h}^{\left(n\right)}=K^{\left(pn\right)}\text{Δ}T+I{\bar{\alpha }}^{\left(pn\right)}\left(T_{h}-\frac{{\bar{\alpha }}^{\left(p\right)}\tau^{\left(p\right)}-{\bar{\alpha }}^{\left(n\right)}\tau^{\left(n\right)}}{{\bar{\alpha }}^{\left(pn\right)}}\text{Δ}T\right)\;-\frac{1}{2}I^{2}R^{\left(pn\right)}\left(1+\frac{R^{\left(p\right)}\beta^{\left(p\right)}+R^{\left(n\right)}\beta^{\left(n\right)}}{R^{\left(pn\right)}}\right). \end{array}$$

Hence we obtain the three DoFs for the leg-pair module as

$$\begin{array}{l} Z_{\text{gen}}^{\left(pn\right)}:=\frac{{\left(V^{\left(pn\right)}/\text{Δ}T\right)}^{2}}{R^{\left(pn\right)}K^{\left(pn\right)}},\\ \tau^{\left(pn\right)}:=\frac{{\bar{\alpha }}^{\left(p\right)}\tau^{\left(p\right)}-{\bar{\alpha }}^{\left(n\right)}\tau^{\left(n\right)}}{{\bar{\alpha }}^{\left(pn\right)}},\\ \beta^{\left(pn\right)}:=\frac{R^{\left(p\right)}\beta^{\left(p\right)}+R^{\left(n\right)}\beta^{\left(n\right)}}{R^{\left(pn\right)}}. \end{array}$$

With this defintion, the heat current has the same form as in single-leg devices:

$$\begin{array}{l} Q_{h}^{\left(pn\right)}=K^{\left(pn\right)}\text{Δ}T+I{\bar{\alpha }}^{\left(pn\right)}\left(T_{h}-\tau^{\left(pn\right)}\text{Δ}T\right)-\frac{1}{2}I^{2}R^{\left(pn\right)}\left(1+\beta^{\left(pn\right)}\right),\\ Q_{c}^{\left(pn\right)}=K^{\left(pn\right)}\text{Δ}T+I{\bar{\alpha }}^{\left(pn\right)}\left(T_{c}-\tau^{\left(pn\right)}\text{Δ}T\right)+\frac{1}{2}I^{2}R^{\left(pn\right)}\left(1-\beta^{\left(pn\right)}\right). \end{array}$$

The power relation is satisfied as

$$Q_{h}^{\left(pn\right)}-Q_{c}^{\left(pn\right)}=I{\bar{\alpha }}^{\left(pn\right)}\left(T_{h}-T_{c}\right)-I^{2}R^{\left(pn\right)}=I\left(V^{\left(pn\right)}-IR^{\left(pn\right)}\right)=I^{2}R_{\text{L}}.$$

#### Design of high efficiency graded legs using Bi_2_Te_3_

This section is related to Section ‘Possible Impact of DoFs’ section in the manuscript. Using *calculated* material properties, we design funtionally graded materials (FGM) composed of Bi_2_Te_3_ to maximize the efficiency. The thermoelectric properties are calculated using the density functional theory (DFT) combined with the Boltzmann transport equation; for detailed computation methods for calculated TEPs, see STAR Methods (Calculation of Thermoelectric Properties of Bi_2_Te_3_).

We calculate the maximum efficiencies of functional gradient layers (FGL) based on Bi_2_Te_3_ for temperature range from 300 K to 600 K. We consider various segmented devices having 1 stage to 8 stages with eight different carrier concentrations $\left(8\times 10^{18},\;1\times 10^{19},\;2\times 10^{19},\;4\times 10^{19},\;8\times 10^{19},\;1\times 10^{20},\;2\times 10^{20}\;{\text{cm}}^{-3}\right)$. We perform high-throughput computation to find the optimal segmented FGL. There are $8^{8}$ possible configurations in total. The temperature distribution inside a single FGL leg device is obtained by using fixed-point iterations of the integral (Equation 7). At the same time, the current density is optimized to find the maximum efficiency; see STAR Methods (Efficiency Computation for Segmented Legs with Contact) for more details. Figure S10 presents the thermoelectric properties calculated by DFT, various segmented structures with its efficiency, and the optimal carrier concentration as a function of position. Figure S11 shows the highest efficiency is obtained for a 5-stage segmented device. For single-stage materials, the maximum efficiency of 10.5% is found at the doping concentration $4\times 10^{19\;}{\text{cm}}^{-3}$. For multi-stage materials, the maximum efficiency is found at the 5 stage with the optimal carrier concentration varying from $8\times 10^{19}\;{\text{cm}}^{-3}$ to 1×10^19^ cm^−3^ as going from hot to cold side.

## Data Availability

Computational algorithms are written in the STAR Methods. The DOIs of the published material data (thermoelectric properties) are listed in the Excel Table S9. Any additional information required to reanalyze the data reported in this paper is available from the lead contact upon request.

## Supplemental information

Document S1. Figures S1–S13 and Table S1–S8

Table S9. Information for 277 published materials, Related to Figure 6 and STAR Methods (277 Published Thermoelectric Property Data) DOIs, reference, available temperature ranges, computed maximum thermoelectric efficiencies, and corresponding DoFs are given.

## Footnotes

Supplemental information can be found online at https://doi.org/10.1016/j.isci.2021.102934.
