# Supplementary material for: Thermoelectric degrees of freedom determining thermoelectric efficiency
Source: iScience. 2021 Aug 5;24(9):102934. doi: 10.1016/j.isci.2021.102934 (PMC8382987; doi:10.1016/j.isci.2021.102934)
Supplement: Document S1. Figures S1–S13 and Table S1–S8 [file mmc1.pdf]

iScience, Volume *24*

Supplemental information

Thermoelectric degrees of freedom determining thermoelectric efficiency

Byungki Ryu, Jaywan Chung, and SuDong Park

Table S1. **Material Groups in the TEP Dataset of 277 Materials, Related to Figure 6 and STAR Methods (277 Published Themo-electric Property Data).** 'Group' and '#mats.' columns represent the group of base material and the number of materials inside the group. For the detailed references, please find the Supporting excel-table Table S7.

**Group**	**#mats**	**Group**	**#mats**
Bi2Te3	59	PbSe	7
PbTe	55	HH	7
SKD	40	SiGe	6
Mg2Si	23	In4Se3	3
GeTe	18	PbS	3
M2Q	14	Oxide	2
SnTe	12	clathrate	2
ABQ2	11	Zintl	1
SnSe	8	etc	6
Total	277		

Table S2. **Basic Information on the 18 Selected Materials, Related to Figures 6 and 7.** Available temperature range from T_c to T_h given in the unit of [K], peak zT, and T of the corresponding peak zT.

ID-#	DOI	Reference	T_h	T_c	peak zT	T of peak zT
1	10.1038/NCHEM.955	Biswas et al. (2011)	817.6	251.0	1.7	800
2	10.1038/nature11439	Biswas et al. (2012)	915.0	302.1	2.2	915
4	10.1038/ncomms9144	Fu et al. (2015a)	1199.8	301.2	1.5	1200
5	10.1002/aenm.201200970	Gelbstein et al. (2013)	713.3	329.4	2	673
6	10.1002/adma.201501030	He et al. (2015a)	997.5	299.0	2.1	1000
9	10.1038/am.2013.86	Hu et al. (2014)	479.4	297.7	1.3	380
10	10.1039/c5ee02979a	Hu et al. (2016)	899.6	306.6	1.8	810
12	10.1126/science.aaa4166	Kim et al. (2015c)	479.7	300.0	1.86	320
17	10.1038/nature09996	Pei et al. (2011g)	847.4	299.9	1.8	850
18	10.1039/c0ee00456a	Pei et al. (2011c)	750.0	300.0	1.4	750
19	10.1126/science.1156446	Poudel et al. (2008)	524.6	299.7	1.4	373
23	10.1073/pnas.1403601111	Wang et al. (2014a)	758.7	321.4	2	773
27	10.1038/nature13184	Zhao et al. (2014)	970.1	302.7	2.6	923
28	10.1126/science.aad3749	Zhao et al. (2015)	773.4	303.5	2	773
34	10.1063/1.3427427	Fan et al. (2010)	512.7	303.0	1.8	316
43	10.3365/eml.2010.12.201	Lee et al. (2010)	572.7	322.9	1.2	373
85	10.1039/c5ee01147g	Wu et al. (2015a)	922.5	302.2	2.3	923
292	10.1063/1.4896520	Zhong et al. (2014)	1019.1	327.4	2.62	1029

Table S3. **Exact and Estimated Maximum Efficiencies of the 18 Selected Materials, Related to Figures 6 and 7.** (a) Exact $\eta_{\max}$ satisfying $\eta(J) \leq \eta_{\max}$. (b) Maximum efficiency estimated from the average parameters $\overline{Q_h}$ and $\overline{Q_c}$. (c)–(f) Estimated maximum efficiencies: (c) using the general maximum efficiency formula $\eta_{\max}^{\text{gen}}$, (d) using the CPM maximum efficiency formula $\eta_{\max}^{\text{cpm}}$, (e) using the modified $[ZT]_{\text{mod}}$ by Min et al., (2014), (f) using the engineering $[ZT]_{\text{eng}}$ and the generic formula by Kim et al. (Kim et al., 2015a, equation [8]).

ID-#	$\eta_{\max}$ at J where $\eta(J) \leq \eta_{\max}$		Maximum efficiency estimation model							
			(c) $\eta_{\max}^{\text{gen}}(Z_{\text{gen}}, \tau, \beta)$ at $\gamma_{\text{opt}}^{\text{gen}}$			(d) $\eta_{\max}^{\text{cpm}}$			(e)	(f)
	(a) exact	(b) CPM part	Z_{gen} τ β	$Z_{\text{gen}}^{(0)}$ $\tau^{(0)}$ $\beta^{(0)}$	$Z_{\text{gen}}^{(0)}$ $\tau_{\text{lin}}^{(0)}$ $\beta_{\text{lin}}^{(0)}$	Z_{gen} - -	$Z_{\text{gen}}^{(0)}$ - -	peak-zT - -	$[ZT]_{\text{mod}}$ - -	$[ZT]_{\text{eng}}$ generic formula
1	13.7%	14.4%	13.8%	14.4%	14.4%	14.5%	15.0%	22.8%	17.6%	12.1%
2	15.9%	16.6%	15.9%	16.2%	16.2%	16.6%	16.8%	25.8%	18.8%	14.7%
4	15.3%	15.8%	15.3%	15.8%	15.8%	15.8%	16.3%	23.3%	18.9%	14.8%
5	12.5%	13.1%	12.6%	12.9%	13.0%	13.1%	13.4%	19.3%	14.1%	12.2%
6	10.5%	11.0%	10.5%	10.7%	10.7%	11.1%	11.1%	26.0%	14.8%	10.2%
9	8.4%	8.4%	8.4%	8.4%	8.4%	8.4%	8.4%	9.0%	8.2%	8.4%
10	13.8%	14.4%	13.8%	14.2%	14.2%	14.4%	14.7%	21.6%	15.3%	12.5%
12	9.1%	9.0%	9.1%	9.1%	9.1%	9.0%	9.0%	11.1%	8.1%	9.3%
17	12.6%	13.2%	12.7%	13.0%	13.0%	13.3%	13.5%	21.3%	14.8%	11.7%
18	10.4%	10.9%	10.4%	10.8%	10.9%	10.9%	11.2%	17.9%	13.2%	9.2%
19	9.9%	9.9%	9.9%	10.0%	10.0%	9.9%	9.9%	11.3%	9.3%	10.0%
23	11.6%	12.2%	11.6%	12.1%	12.1%	12.2%	12.5%	19.2%	14.1%	10.2%
27	7.1%	7.1%	7.1%	7.1%	7.1%	7.1%	7.1%	28.3%	5.5%	7.8%
28	16.2%	16.7%	16.2%	16.9%	17.0%	16.7%	17.3%	21.0%	18.3%	14.8%
34	10.1%	10.0%	10.1%	10.1%	10.1%	10.0%	10.0%	12.1%	8.6%	10.2%
43	8.2%	8.1%	8.2%	8.2%	8.2%	8.1%	8.1%	10.2%	6.9%	8.3%
85	17.6%	18.4%	17.6%	18.1%	18.1%	18.5%	18.8%	25.4%	17.8%	16.5%
292	14.3%	14.9%	14.3%	14.9%	14.9%	14.9%	15.4%	27.1%	19.4%	14.2%

Table S4. **Relative Errors in Estimating the Maximum Efficiencies of the 18 Selected Materials, Related to the Figure 6 and Table S3.**

ID-#	Relative Errors of maximum efficiency estimation models									
	at J where $\eta(J) \le \eta_{\max}$		(c) $\eta^{\text{gen}}_{\max}(Z_{\text{gen}}, \tau, \beta)$ at $\gamma^{\text{gen}}_{\text{opt}}$			(d) $\eta^{\text{cpm}}_{\max}$			(e)	(f)
	(a) exact	(b) CPM part	Z_{gen} τ β	$Z^{(0)}_{\text{gen}}$ $\tau^{(0)}$ $\beta^{(0)}$	$Z^{(0)}_{\text{gen}}$ $\tau^{(0)}_{\text{lin}}$ $\beta^{(0)}_{\text{lin}}$	Z_{gen} - -	$Z^{(0)}_{\text{gen}}$ - -	peak-zT - -	$[ZT]_{\text{mod}}$ - -	$[ZT]_{\text{eng}}$ generic formula
1	0	5.18%	0.13%	4.73%	5.14%	5.46%	9.20%	65.91%	27.83%	-12.00%
2	0	4.72%	0.03%	2.19%	1.90%	4.84%	6.16%	62.76%	18.50%	-6.97%
4	0	3.31%	0.08%	3.60%	3.60%	3.45%	6.46%	52.62%	23.57%	-3.47%
5	0	4.51%	0.09%	3.15%	3.68%	4.65%	6.53%	53.97%	12.36%	-2.98%
6	0	4.75%	-0.03%	1.28%	1.66%	4.85%	5.33%	146.74%	40.22%	-3.52%
9	0	-0.12%	0.00%	0.57%	0.62%	-0.12%	0.42%	7.67%	-2.45%	0.36%
10	0	4.13%	0.01%	2.27%	2.54%	4.23%	5.84%	55.79%	10.34%	-9.91%
12	0	-0.98%	0.00%	-0.12%	-0.17%	-0.98%	-1.13%	21.88%	-11.55%	1.42%
17	0	4.73%	0.09%	2.80%	3.06%	4.87%	6.76%	68.75%	17.12%	-7.09%
18	0	4.36%	0.08%	4.01%	4.43%	4.56%	7.62%	72.47%	26.77%	-11.94%
19	0	-0.39%	0.00%	0.74%	0.78%	-0.39%	0.32%	13.88%	-6.03%	1.37%
23	0	4.57%	0.07%	3.75%	4.04%	4.76%	7.39%	65.60%	20.96%	-12.16%
27	0	0.07%	0.00%	0.65%	0.78%	0.07%	0.68%	300.36%	-21.76%	11.04%
28	0	2.99%	0.11%	4.68%	4.97%	3.19%	6.89%	29.87%	13.05%	-8.52%
34	0	-1.10%	0.00%	-0.09%	-0.25%	-1.10%	-1.24%	20.09%	-14.55%	1.16%
43	0	-0.96%	0.00%	0.03%	0.00%	-0.95%	-0.94%	24.58%	-14.97%	1.68%
85	0	4.87%	0.03%	2.69%	2.89%	5.01%	6.75%	44.27%	1.15%	-6.38%
292	0	4.57%	0.33%	4.69%	4.40%	4.81%	8.21%	90.20%	36.15%	-0.62%
std	0	2.48%	0.08%	1.73%	1.83%	2.56%	3.65%	66.94%	18.51%	6.17%

Table S5. **Exact Value and One-Shot Approximation of Thermoelectric Degees of Freedom of the 18 Slected Materials, Related to Figure 6(A–C).** Exact values are computed at the maximum efficiency condition.

ID-#	Z_{gen}	$Z_{\text{gen}}^{(0)}$	τ	$\tau^{(0)}$	$\tau_{\text{lin}}^{(0)}$	β	$\beta^{(0)}$	$\beta_{\text{lin}}^{(0)}$
1	0.00152	0.00159	-0.25297	-0.20676	-0.19151	0.19195	0.19929	0.20171
2	0.00176	0.00179	-0.18571	-0.15244	-0.16574	0.06779	0.07428	0.08775
4	0.00104	0.00109	-0.16392	-0.14073	-0.13966	0.19667	0.20261	0.19755
5	0.00224	0.00230	-0.22680	-0.16824	-0.14485	0.09445	0.10529	0.10703
6	0.00084	0.00085	-0.25318	-0.20771	-0.18948	0.02699	0.02813	0.03423
9	0.00291	0.00293	-0.01863	-0.01654	-0.01321	0.13533	0.13562	0.13546
10	0.00148	0.00151	-0.19199	-0.16111	-0.15197	0.10184	0.10697	0.11507
12	0.00334	0.00333	0.02950	0.03172	0.02979	0.17663	0.17773	0.17147
17	0.00143	0.00147	-0.23142	-0.18911	-0.17825	0.10859	0.11244	0.11338
18	0.00136	0.00142	-0.27077	-0.21423	-0.18997	0.16723	0.17172	0.15859
19	0.00281	0.00284	-0.01500	-0.01342	-0.01126	0.18903	0.19001	0.19109
23	0.00168	0.00174	-0.25361	-0.19424	-0.17773	0.13782	0.14247	0.12988
27	0.00051	0.00051	0.08250	0.08629	0.05964	-0.37853	-0.38177	-0.22354
28	0.00247	0.00263	-0.15423	-0.11828	-0.09886	0.21719	0.22529	0.17537
34	0.00316	0.00315	0.03336	0.03596	0.02727	0.16434	0.16555	0.16269
43	0.00187	0.00187	0.02779	0.02892	0.02906	0.18590	0.18699	0.17628
85	0.00205	0.00211	-0.17877	-0.14647	-0.13887	0.07940	0.09546	0.09146
292	0.00132	0.00138	-0.21142	-0.16586	-0.16984	0.17801	0.18705	0.15349

Table S6. **Statistics on Relative Errors of the Maximum Efficiency Estimation Formula, Related to Figure 6.** Average of RE (Avg.), root mean square of RE (RMSRE), absolute mean of relative error (L^1), minimum of the RE [min(RE)], maximum of the RE [max(RE)], and maximum of the absolute RE (L^∞) are estimated for 277 materials for thermoelectric power generator working at their available temperature range.

$\eta_{\max}$ estimation model	Avg.	RMSRE	L^1	min(RE)	max(RE)	L^∞
$\eta_{\max}^{\mathrm{cpm}}$ using peak-zT	224%	1800%	224%	3.76%	27449%	27449%
$\eta_{\max}^{\mathrm{cpm}}$ using $[ZT]_{\mathrm{mod}}$ by Min et al. (2004)	9.6%	24.4%	16.0%	-74.0%	158.1%	158.1%
$\eta_{\max}$ using generic forumula with $[ZT]_{\mathrm{eng}}$	-2.169%	7.164%	4.101%	-30.62%	53.68%	53.68%
$\eta_{\max}^{\mathrm{gen}}(Z_{\mathrm{gen}}^{(0)}, 0, 0) = \eta_{\max}^{\mathrm{cpm}}(Z_{\mathrm{gen}}^{(0)})$	2.293%	3.364%	2.412%	-2.481%	9.96%	9.959%
$\eta_{\max}^{\mathrm{gen}}(Z_{\mathrm{gen}}^{(0)}, \tau_{\mathrm{lin}}^{(0)}, \beta_{\mathrm{lin}}^{(0)})$	1.189%	1.889%	1.300%	-2.491%	5.77%	5.767%
$\eta_{\max}^{\mathrm{gen}}(Z_{\mathrm{gen}}^{(0)}, \tau^{(0)}, \beta^{(0)})$	1.118%	1.772%	1.210%	-1.920%	5.45%	5.451%
$\eta_{\max}^{\mathrm{gen}}(Z_{\mathrm{gen}}, 0, 0) = \eta_{\max}^{\mathrm{cpm}}(Z_{\mathrm{gen}})$	1.415%	2.079%	1.549%	-1.783%	5.80%	5.803%
CPM part of $\eta_{\max}$	1.377%	2.016%	1.512%	-1.797%	5.49%	5.488%
$\eta_{\max}^{\mathrm{gen}}(Z_{\mathrm{gen}}, \tau_{\mathrm{lin}}, \beta_{\mathrm{lin}})$	0.336%	0.557%	0.374%	-0.904%	1.88%	1.878%
$\eta_{\max}^{\mathrm{gen}}(Z_{\mathrm{gen}}, \tau, 0)$	-0.256%	0.444%	0.350%	-1.469%	1.17%	1.469%
$\eta_{\max}^{\mathrm{gen}}(Z_{\mathrm{gen}}, \tau, \beta)$	0.016%	0.097%	0.035%	-0.606%	1.15%	1.154%
$\eta_{\max}$ (exact, numerical)	0	0	0	0	0	0

TABLE S7. **Kendall Rank Correlation Coefficient between Exact Efficiency and Estimated η_{max} (or Z_{gen}) for 277 Published Materials, with various Working Temperature Range, Related to Figures 6(D,E), and STAR Methods (Kendall Rank Correlation Coefficients of Maximum Efficiency Estimation Methods).** The 'autoTcTh' means the available temperature range of materials.

Efficiency and figure of merit model		Working temperature range (T_c-T_h)			
x	y	300-400	300-600	300-900	autoTcTh
η_{max}	$\eta_{\text{max}}^{\text{gen}}(Z_{\text{gen}}, \tau, \beta)$	0.9998	0.9999	0.9975	0.9993
	$\eta_{\text{max}}^{\text{gen}}(Z_{\text{gen}}, \tau, 0)$	0.9996	0.9976	0.9987	0.9959
	$\eta_{\text{max}}^{\text{gen}}(Z_{\text{gen}}, 0, 0) = \eta_{\text{max}}^{\text{cpm}}(Z_{\text{gen}})$	0.9992	0.9910	0.9749	0.9837
	$\eta_{\text{max}}^{\text{gen}}(Z_{\text{gen}}^{(0)}, \tau^{(0)}, \beta^{(0)})$	0.9993	0.9910	0.9749	0.9846
	$\eta_{\text{max}}^{\text{gen}}(Z_{\text{gen}}^{(0)}, \tau_{\text{lin}}^{(0)}, \beta_{\text{lin}}^{(0)})$	0.9993	0.9910	0.9724	0.9838
	$\eta_{\text{max}}^{\text{gen}}(Z_{\text{gen}}^{(0)}, 0, 0) = \eta_{\text{max}}^{\text{cpm}}(Z_{\text{gen}}^{(0)})$	0.9988	0.9858	0.9699	0.9731
	η_{max} (generic) using $[ZT]_{\text{eng}}$	0.9956	0.9617	0.9060	0.9400
	$\eta_{\text{max}}^{\text{cpm}}$ with $[ZT]_{\text{mod}}$ by Min and Rowe	0.9722	0.8797	0.7657	0.8090
	$\eta_{\text{max}}^{\text{cpm}}$ with peak-zT (peak-zT)	0.9036	0.7348	0.4900	0.5618
	$\eta_{\text{max}}^{\text{cpm}}$ with $z_h T_h$ (local-zT_h)	0.8954	0.6959	0.4411	0.5391
	Z_{gen}	0.9992	0.9910	0.9749	0.4419
	$Z_{\text{gen}} T_{\text{mid}}$	0.9992	0.9910	0.9749	0.5746
	$Z_{\text{gen}} \Delta T$	0.9992	0.9910	0.9749	0.9294
Number of samples		267	217	57	277
Number of combinations		35511	23436	1596	38226

Table S8. **Comparison of Top Ranks in Order of Exact Maximum Efficiency and Estimation Parameters, Related to Figure 7A, Equations (4) and (19), and STAR Methods (Estimation of Efficiency Rank using $Z_{\mathrm{gen}}^{(0)}$).** The top-rank-preserving probability means the ratio of the number of correct top ranks predicted by the estimation parameter, to the total number of top ranks. The 18 candidate materials in Table S2 are used to generate 5-stage segmented legs. Each stage of the leg has the same cross sectional area $1\,\mathrm{mm}^2$ and the same length $1/5\,\mathrm{mm}$ (total length is set to $1\,\mathrm{mm}$). The hot- and cold-side temperatures are $T_h = 900$ K and $T_c = 300$ K.

Rank		Top-Rank-Preserving Probability		
percent	numbers	$\eta_{\mathrm{max}}^{\mathrm{gen}}(Z_{\mathrm{gen}}, 0, 0)$	$\eta_{\mathrm{max}}^{\mathrm{gen}}(Z_{\mathrm{gen}}^{(0)}, 0, 0)$	$\eta_{\mathrm{max}}^{\mathrm{gen}}(Z_{\mathrm{gen}}^{(0)}, 0, 0)$
Top 0.1%	<1891	87%	73%	73%
Top 1%	<18897	90%	84%	82%
Top 2%	<37792	93%	88%	86%
Top 4%	<75584	94%	89%	90%
100%	1,889,568	100%	100%	100%

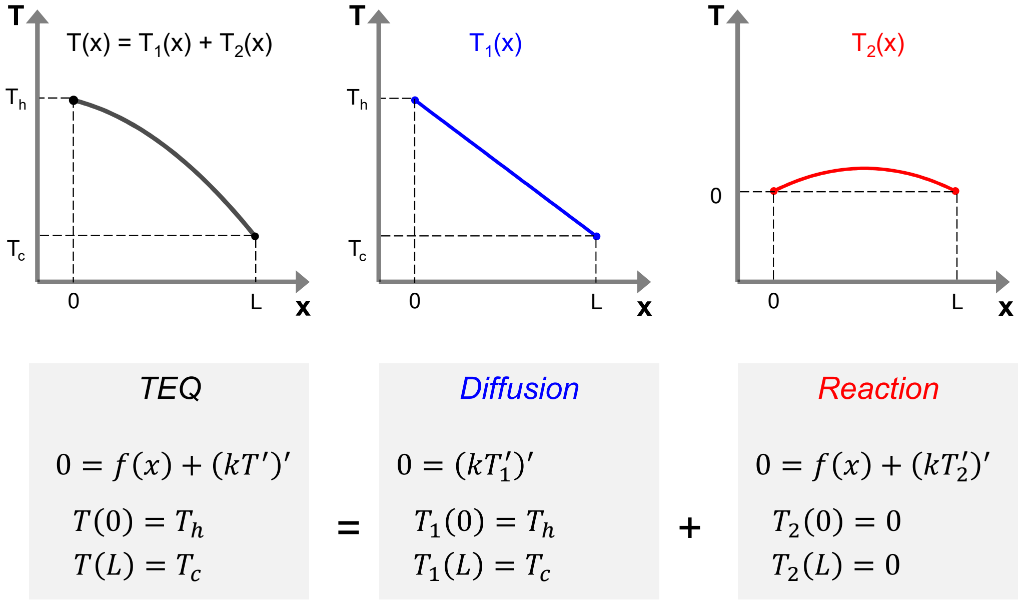

FIGURE S1. **Temperature decomposition for Temperature Solution of 1D Single Leg, Related to STAR Methods (Integral Equations of $T(x)$ and $\frac{dT}{dx}(x)$).**

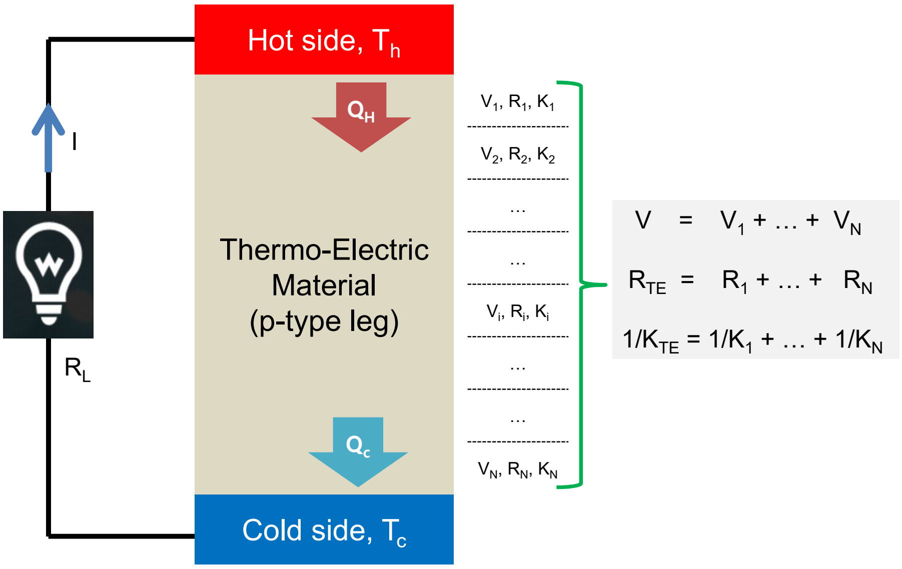

FIGURE S2. **Structure of Conventional Thermoelectric Power Devices, Related to Equation (8).** For simplicity, we draw only an uni-leg with p-type materials where electric current flows from hot to cold side. Since the electric current and heat current flow though the leg, the electrical and thermal resistance of the leg should be considered as the sum of an infinitesimal serial circuit. Thus, the voltage V and the resistance R should be the sum of component voltages and resistances respectively. In the case of thermal conduction, the inverse of thermal conductivity should be used for thermal circuit parameter.

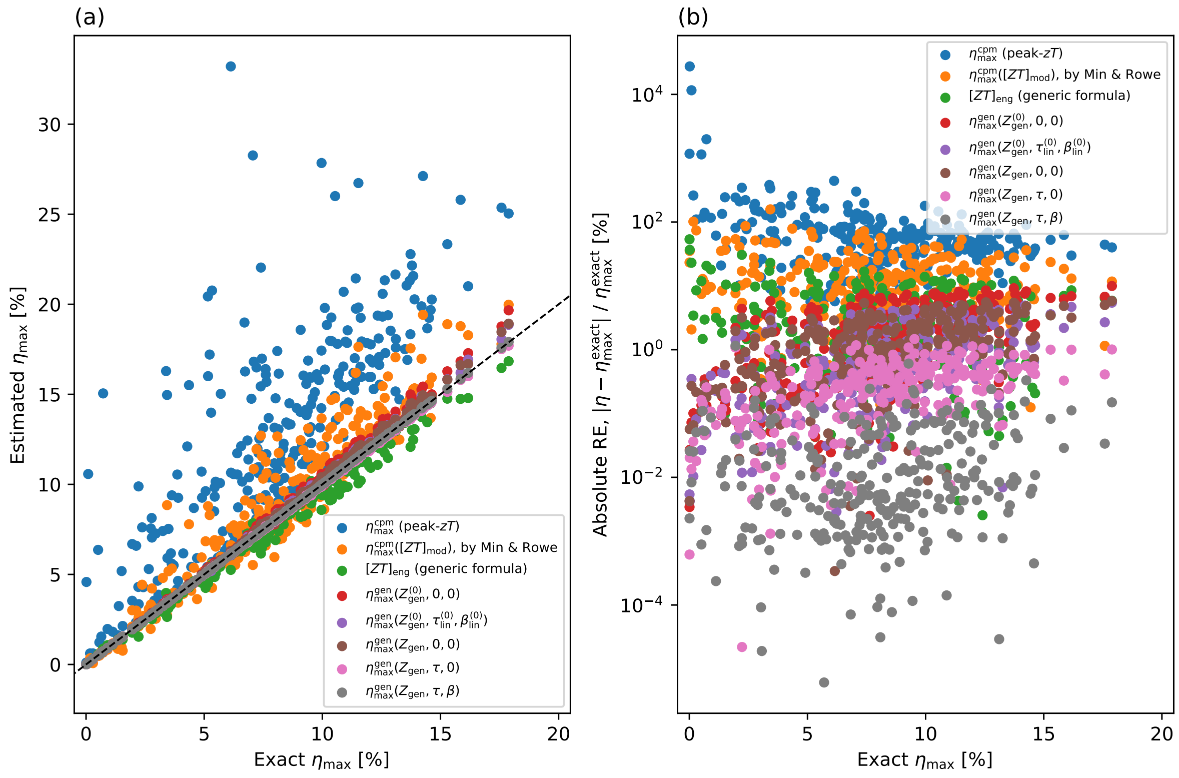

FIGURE S3. **Efficiency Estimation using $\eta^{\text{gen}}_{\max}(Z_{\text{gen}}, \tau, \beta)$ for 277 Published Materials, Related to Figures 6 and 7, Equation (16), and Table S7.** Efficiency estimation for 277 materials using the formula $\eta^{\text{gen}}_{\max}(Z_{\text{gen}}, \tau, \beta)$ are compared to other efficiency estimation model based on $\eta^{\text{cpm}}_{\max}$ with the peak zT and the modified $[ZT]_{\text{mod}}$ by Min et al. (Min et al. (2004)). We also test the engineering $[ZT]_{\text{eng}}$ with generic formula (Kim et al., 2015a, equation [8]). In use of $\eta^{\text{gen}}_{\max}(Z_{\text{gen}}, \tau, \beta)$, there are five options: using (i) exact $Z_{\text{gen}}, \tau, \beta$, (ii) $Z^{(0)}_{\text{gen}}, \tau^{(0)}, \beta^{(0)}$, (iii) $Z^{(0)}_{\text{gen}}, \tau^{(0)}_{\text{lin}}, \beta^{(0)}_{\text{lin}}$, (iv) exact Z_{gen} only, $\tau = 0$, $\beta = 0$ (Z), (v) $Z^{(0)}_{\text{gen}}$ only, $\tau = 0$, $\beta = 0$ (Z0). (a) Comparison of the exact maximum efficiency and the estimations. (b) Relative error (RE) of the estimations (absolute error divided by the exact maximum efficiency). The peak zT is malfunctioning in efficiency prediction; the relative error can be over 100%. On the other hand, the formula $\eta^{\text{gen}}_{\max}(Z_{\text{gen}}, \tau, \beta)$ has small relative error; even the simplest formula with $Z^{(0)}_{\text{gen}}, \tau^{(0)}_{\text{lin}}, \beta^{(0)}_{\text{lin}}$ has the standard error (=root mean square of relative errors) less than 2%.

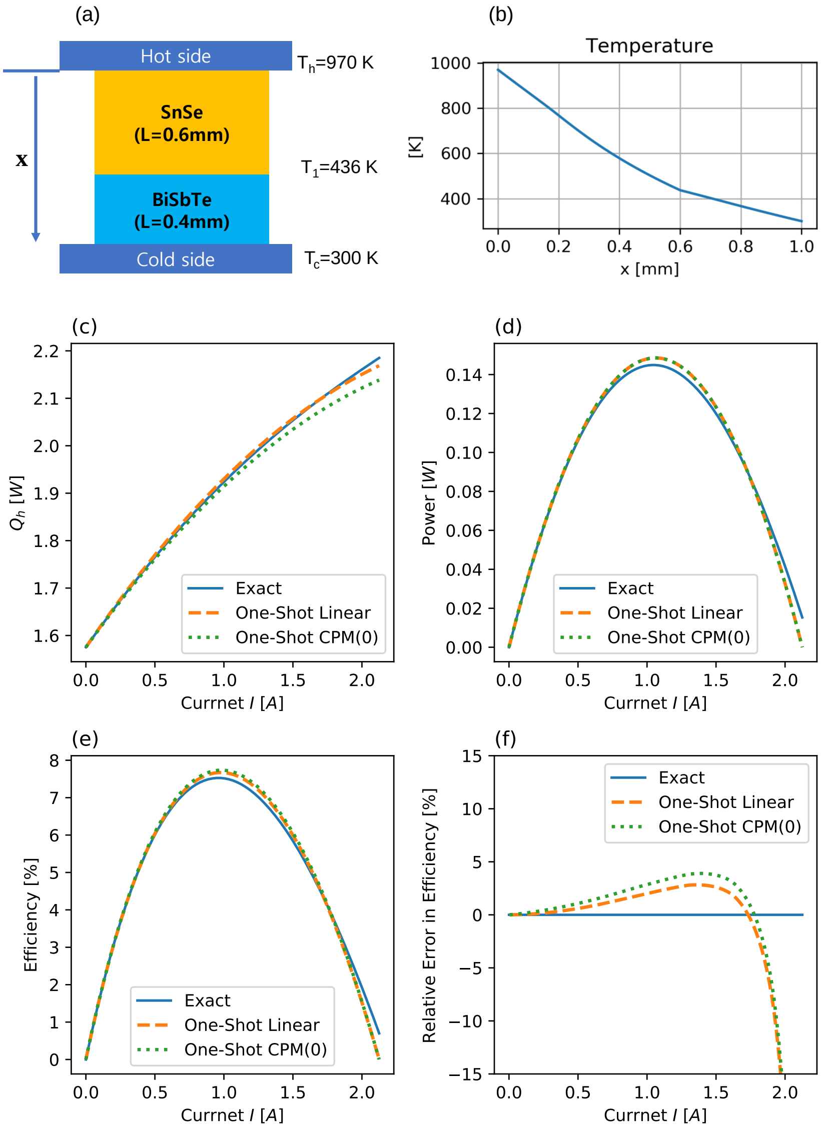

FIGURE S4. **Accuracy of One-Shot Approximations for a Two-Stage Segmented Leg, Related to Equations (18,19) and STAR Methods (Accuracy of the One-Shot Approximation for Segmented Leg).** The thermoelectric performances of a two-stage segmented leg having $3 \times 3\text{mm}^2$ cross section and 2mm leg length, predicted by the one-shot approximations. The numerical exact values are computed by fixed-point iteration and the one-shot values are computed using the one-shot linear approximation (using Q_h with $K = K^{(0)}$, $\overline{\alpha} = \overline{\alpha}^{(0)}$, $\tau = 0$, $R = R^{(0)}$, $\beta = 0$). (a) The geometry of the segmented leg: SnSe and BiSbTe are used for hot- and cold-side materials. $T_h = 970$ K and $T_c = 300$ K are used. (b) Exact temperature distribution obtained by solving the integral equation of T (see equation (7) in the article) with fixed-point iteration. (c) Heat current at the hot side, (d) Power delivered to the outside, (e) Efficiency, and (f) Relative errors of the one-shot approximations compared to the exact efficiency.

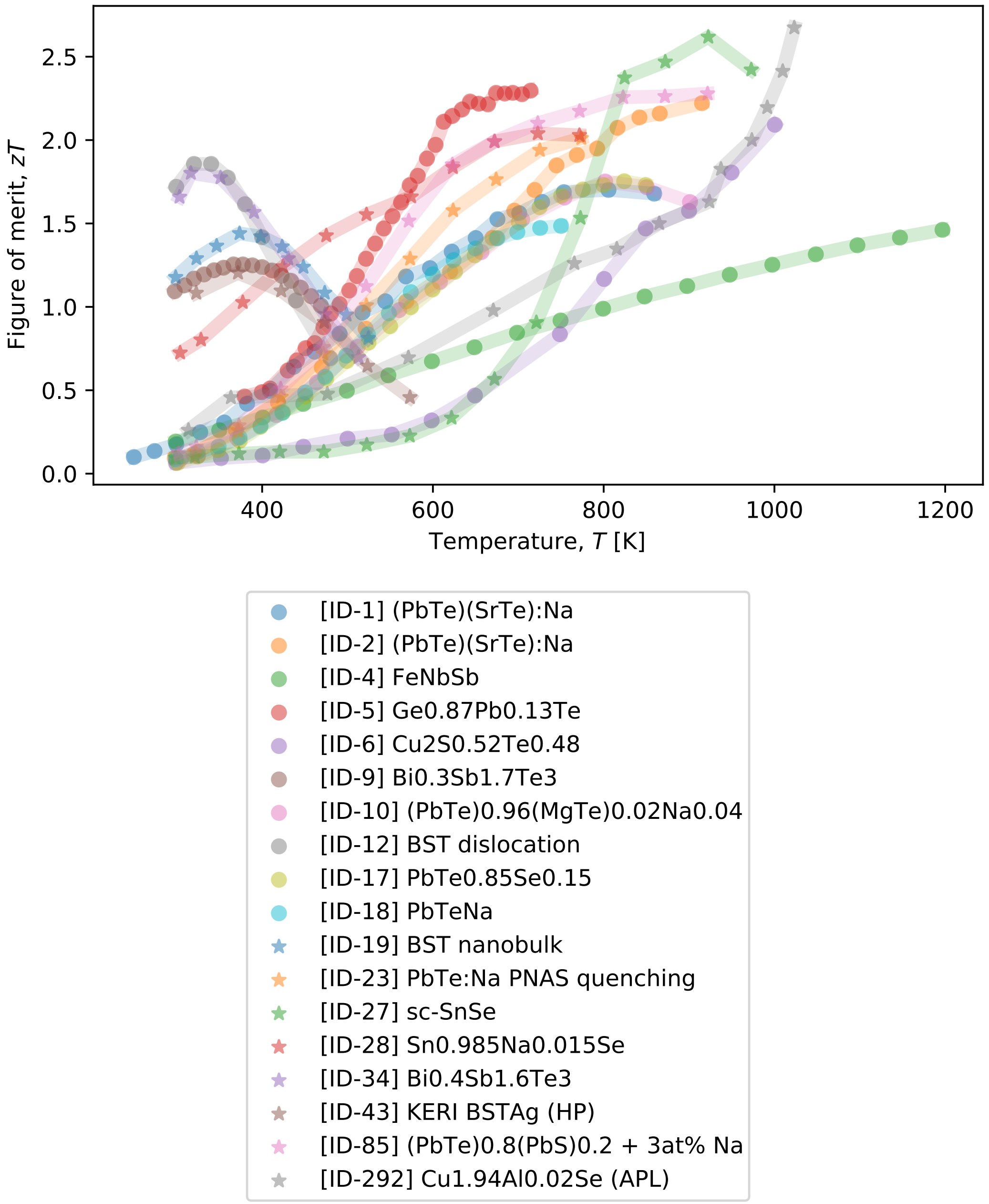

FIGURE S5. **Figures of merit zT for 18 Selected Materials, Related to Figures 6 and 7.**

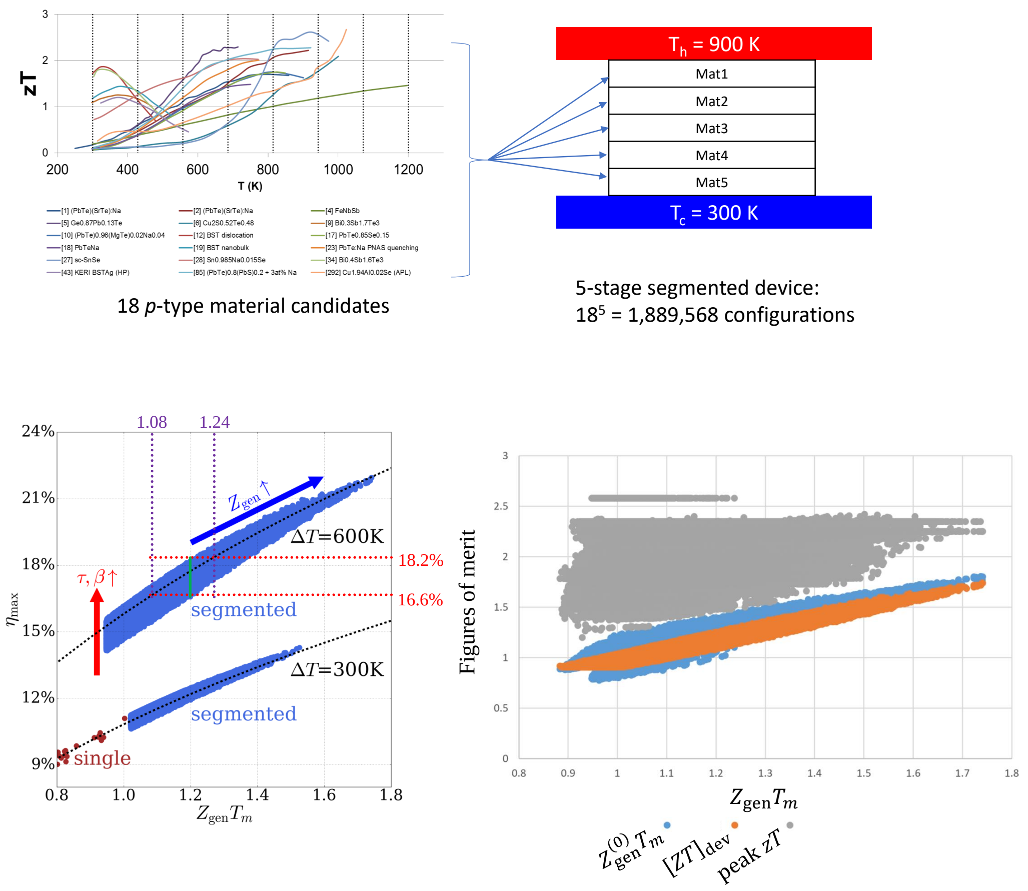

Figure S6. **Impact of DoFs on $\eta_{\max}$ of Segmented Legs, Related to Figure 7 and STAR Methods (Impact of Gradient Parameters τ and β in Segmented Legs).** Using the 18 p-type candidate materials in Table S2, we construct 5-stage segmented legs. When the segmented leg is operating under $T_h = 900$ K and $T_c = 300$ K, the efficiency can reach a high efficiency of 21.9%. When $Z_{\text{gen}}T_m = 1.2$, the efficiency varies by a factor of 10%, which corresponds to the $[ZT]_{\text{dev}}$ change of 0.17.

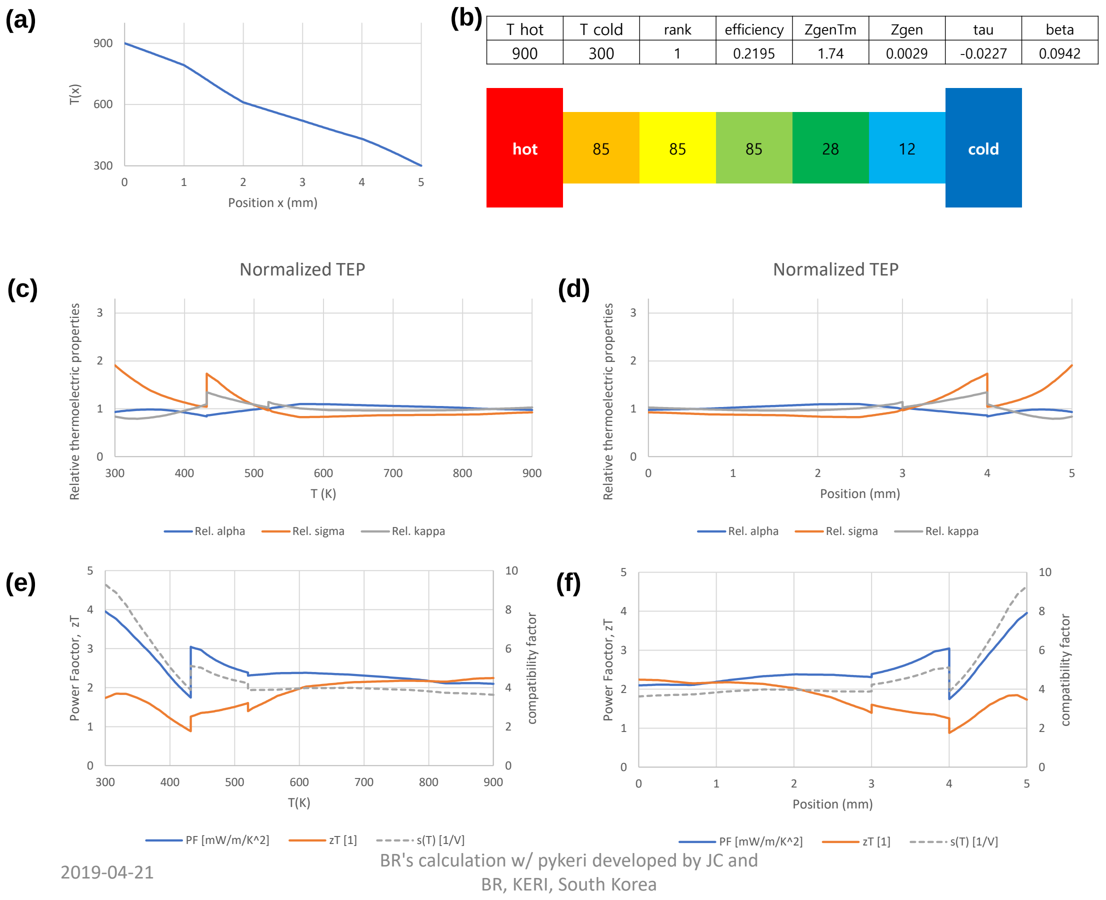

T hot	T cold	rank	efficiency	ZgenTm	Zgen	tau	beta
900	300	1	0.2195	1.74	0.0029	-0.0227	0.0942

Figure S7. **Thermoelectric Property Analysis for the Best Segmented Leg among 18^5 Configurations, Related to Figure 7 and STAR Methods (Impact of Gradient Parameters τ and β in Segmented Legs).** (a) Temperature distribution $T(x)$. (b) Structure of the segmented legs. (c) Normalized thermoelectric properties on $T(x)$. (d) Normalized thermoelectric properties on the position x. (e) Power factor, zT, and compatibility factor $s(T)$ on $T(x)$. (f) Power factor, zT, and compatibility factor $s(T)$ on the position x.

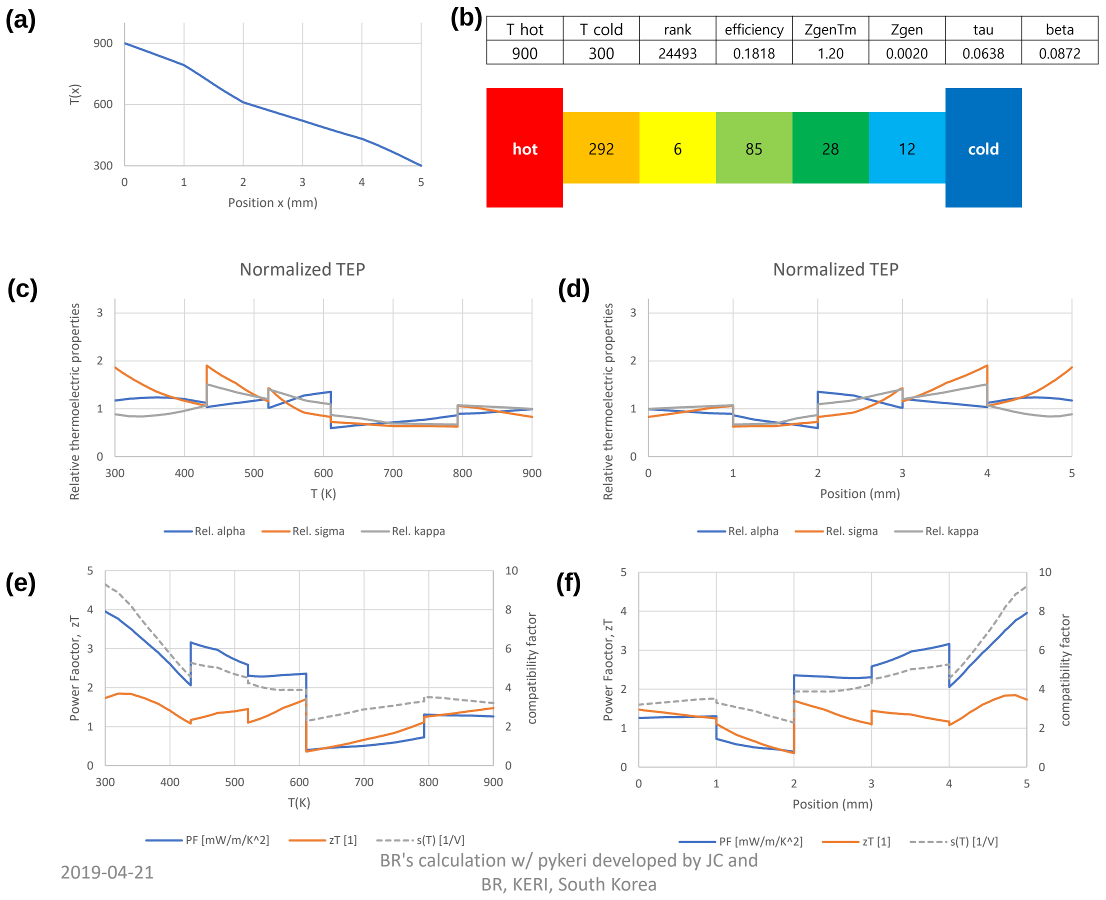

Figure S8. **Thermoelectric Property Analysis for the Segmented Leg of Rank 24493 among 18^5 Configurations, Related to Figure 7 and STAR Methods (Impact of Gradient Parameters τ and β in Segmented Legs).** (a) Temperature distribution $T(x)$. (b) Structure of the segmented legs. (c) Normalized thermoelectric properties on $T(x)$. (d) Normalized thermoelectric properties on the position x. (e) Power factor, zT, and compatibility factor $s(T)$ on $T(x)$. (f) Power factor, zT, and compatibility factor $s(T)$ on the position x.

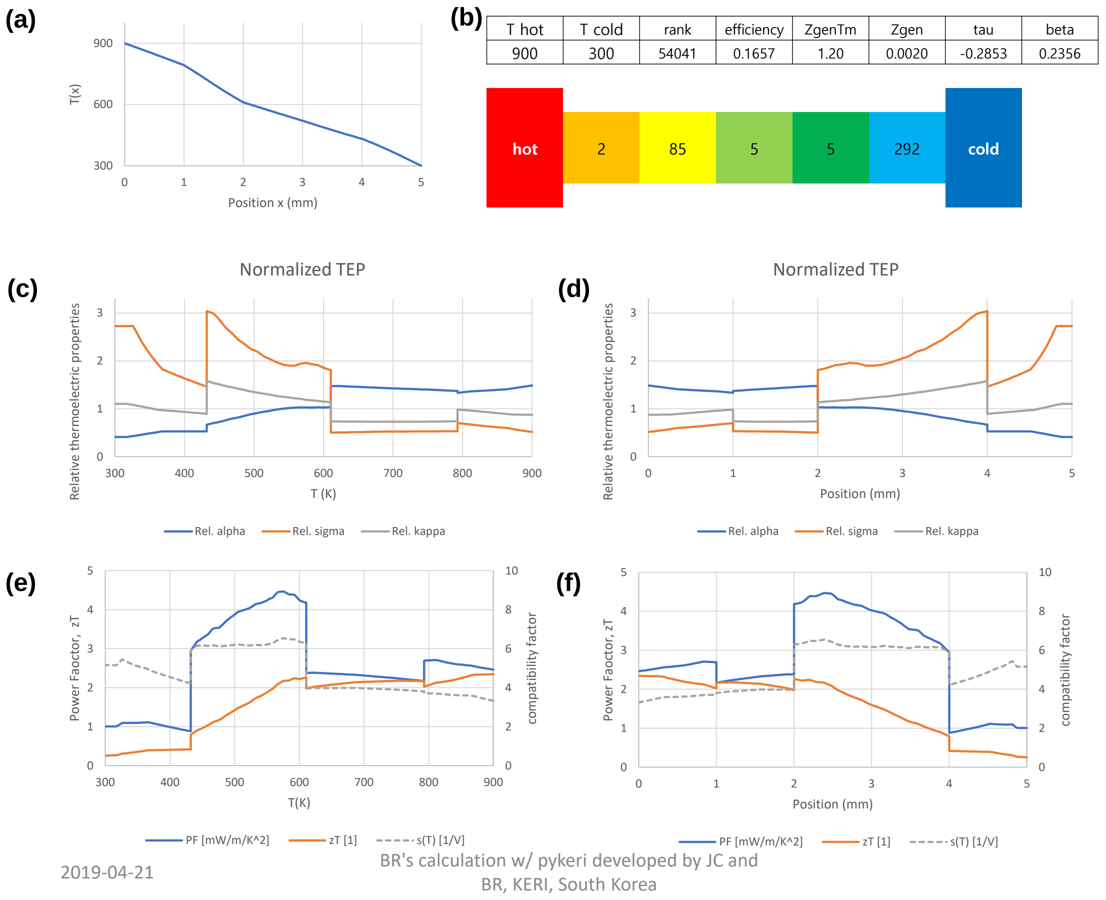

Figure S9. **Thermoelectric Property Analysis for the Segmented Leg of Rank 54041 among 18^5 Configurations, Related to Figure 7 and STAR Methods (Impact of Gradient Parameters τ and β in Segmented Legs).** (a) Temperature distribution $T(x)$. (b) Structure of the segmented legs. (c) Normalized thermoelectric properties on $T(x)$. (d) Normalized thermoelectric properties on the position x. (e) Power factor, zT, and compatibility factor $s(T)$ on $T(x)$. (f) Power factor, zT, and compatibility factor $s(T)$ on the position x.

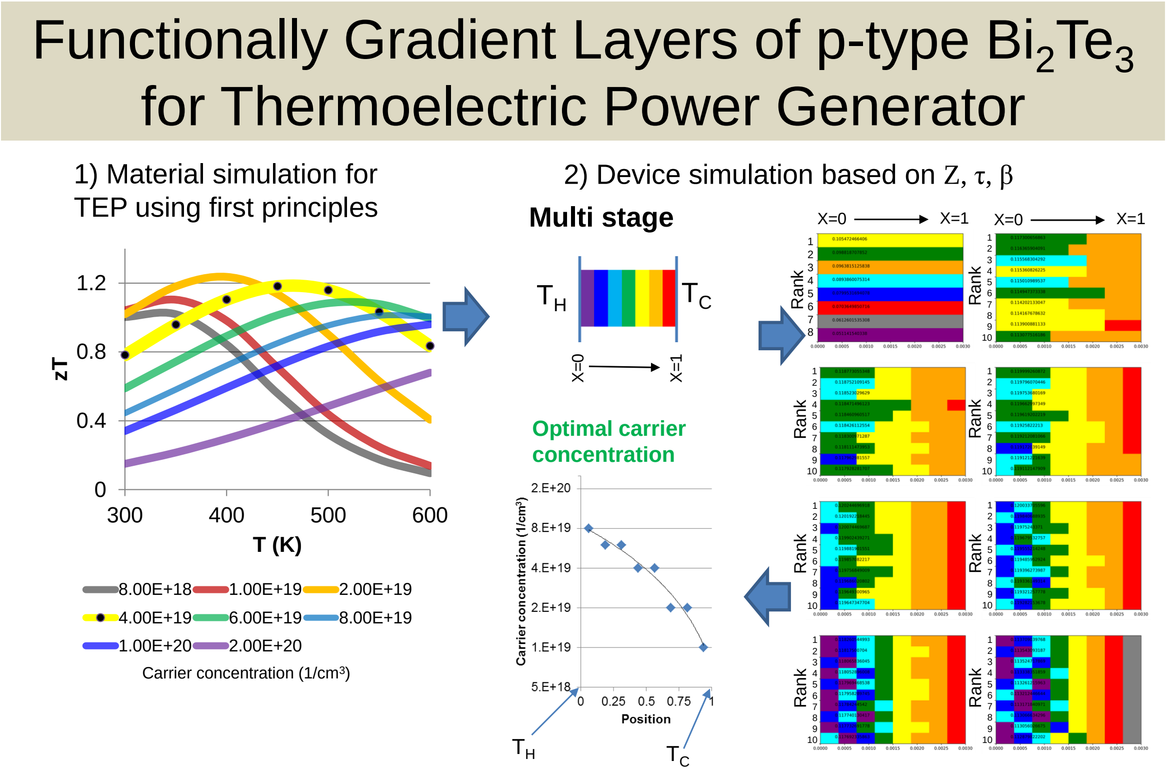

FIGURE S10. **Design process of functionally graded *p*-type** Bi_2Te_3 **materials for thermoelectric power generator and its result, Related to STAR Methods (Design of High Efficiency Graded Legs using** Bi_2Te_3**).** The temperature range from 300 K to 600 K is considered. High-throughput computation of efficiency is performed to search the optimal carrier doping concentration. (Left) Thermoelectric properties of Bi_2Te_3 calculated by DFT. (Middle Top) Schematic structure of segmented devices; different color means different doping concentration. (Right) Top 10 segmented structures when the number of stage (number of segmentation of equal length) is fixed; 1 to 8 stages are considered. (Middle Bottom) Optimal carrier doping concentration having the highest efficiency.

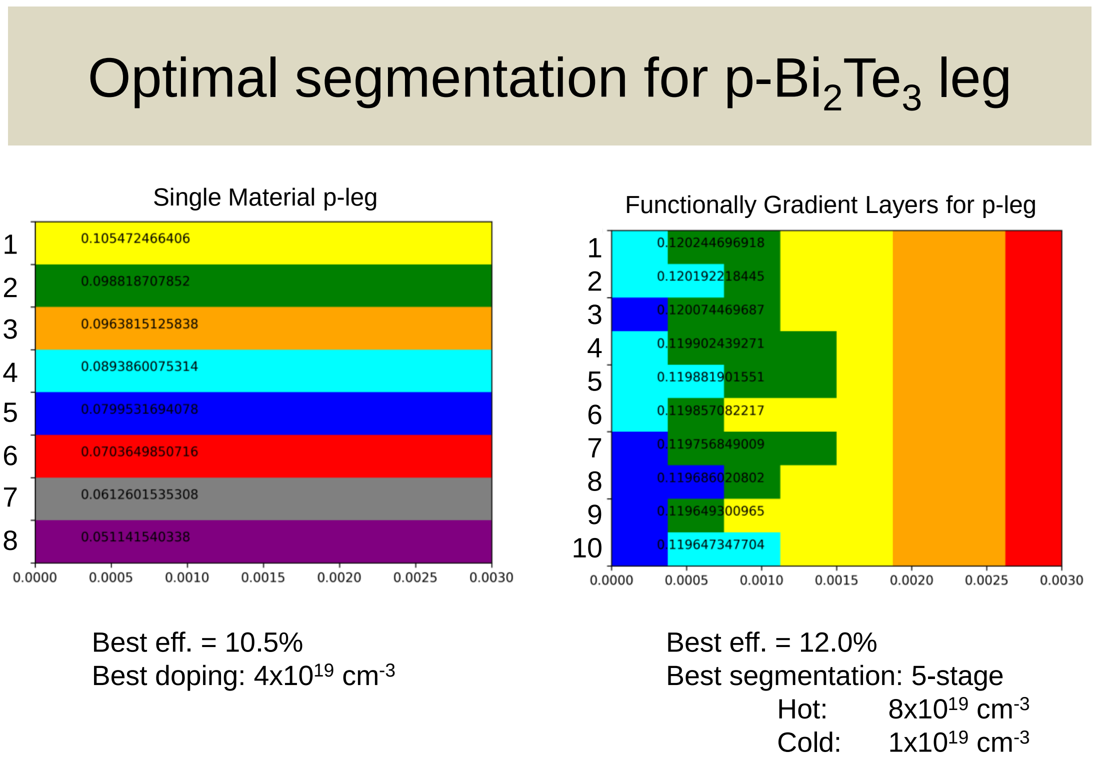

Figure S11. **Top-10 High-Efficiency Segmented Structures with *p*-type Bi_2Te_3 Materials, Related to STAR Methods (Design of High Efficiency Graded Legs using Bi_2Te_3).** (Left) No segmentation. (Right) 5-stage segmentation. Each color represents a distinct material. The best structure is shown in the first row. The yellow is the best for no segmentation. The 1cyan-2green-2yellow-2orange-1red segmented structure in the first row of the right figure is optimal among the 8^8 configurations with the highest efficiency of 12.0%.

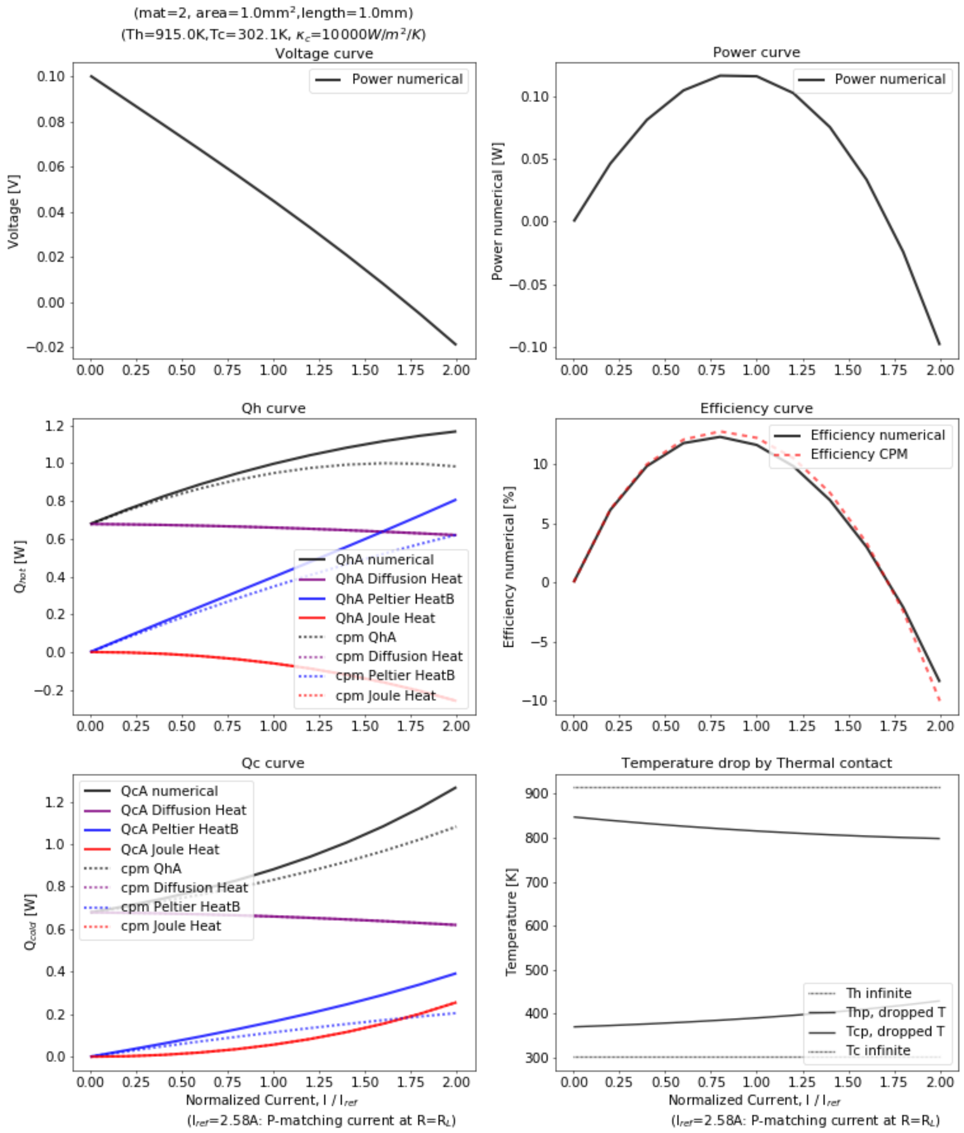

FIGURE S12. **Computed Thermoelectric Performances of Single Leg Device Using ID-2, Related to Section 'Electrical and Thermal Engineering under Contact Resistance'.** Parameters and values are computed using a numerical solver, exact DoF values at every I Points, and one-shot DoF values. In x-axis, the maximum power current is set to 1. The thermal contact resistance of $10,000\ \mathrm{W/m^2/K}$ is used for hot and cold side contacts. The Z_{gen}, τ, and β are computed at the maximum power current condition. Then, using them, the thermoelectric properties at arbitrary I is analytically calculated.

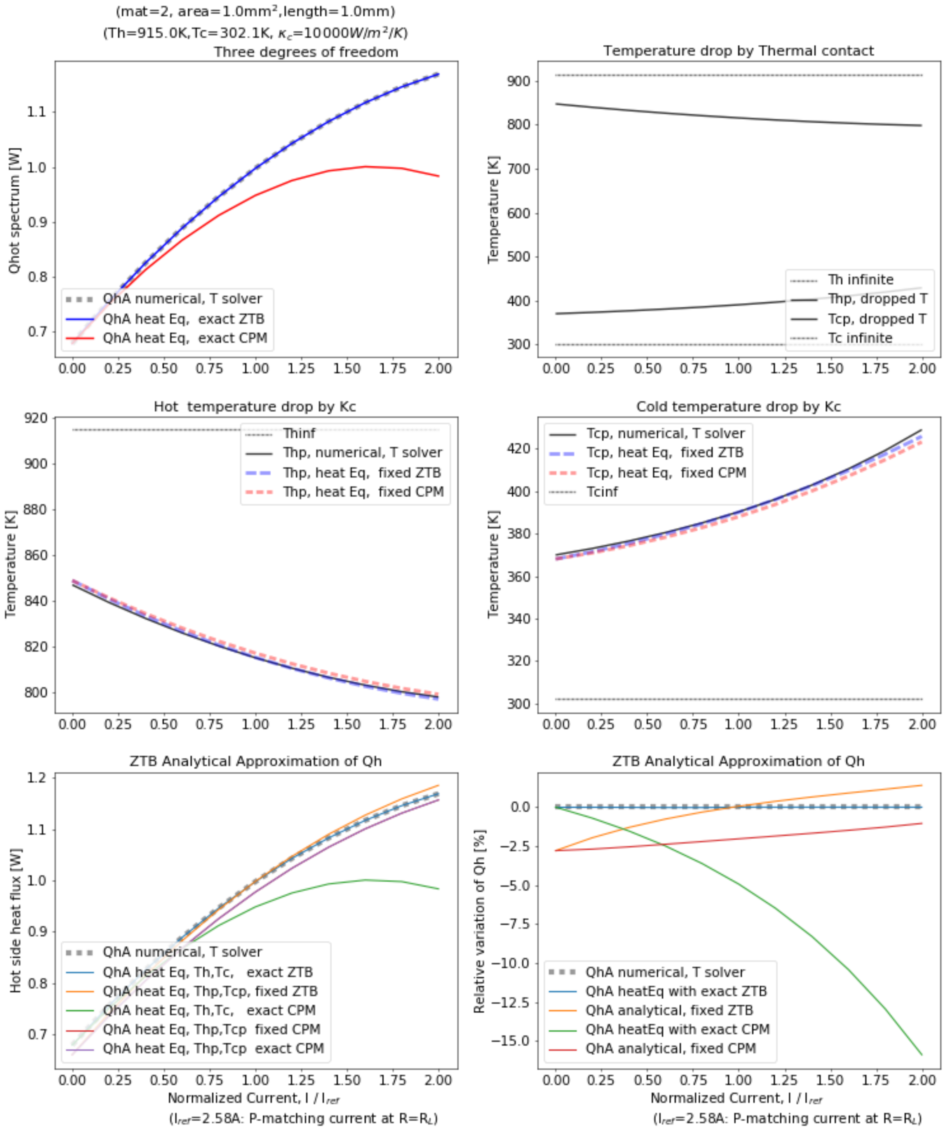

Figure S13. **Computed Temperature of Single Leg Device Using ID-2, Related to Section 'Electrical and Thermal Engineering under Contact Resistance'** Parameters and values are computed using a numerical solver, exact DoF values at every I Points, and one-shot DoF values. In x-axis, the maximum power current is set to 1. The thermal contact resistance of $10,000$ W/m^2/K is used for hot and cold side contacts. The Z_{gen}, τ, and β are computed at the maximum power current condition. Then, using them, the thermoelectric properties at arbitrary I is analytically calculated.
